# Developing predictive models for µ opioid receptor binding using machine learning and deep learning techniques

**DOI:** 10.3389/ebm.2025.10359

**Published:** 2025-03-19

**Authors:** Jie Liu, Jerry Li, Zoe Li, Fan Dong, Wenjing Guo, Weigong Ge, Tucker A. Patterson, Huixiao Hong

**Affiliations:** ^1^ U.S. Food and Drug Administration, National Center for Toxicological Research, Jefferson, AR, United States; ^2^ Department of Computer Science, Rice University, Houston, TX, United States

**Keywords:** μ opioid receptor, binding activity, machine learning, deep learning, predictive model

## Abstract

Opioids exert their analgesic effect by binding to the µ opioid receptor (MOR), which initiates a downstream signaling pathway, eventually inhibiting pain transmission in the spinal cord. However, current opioids are addictive, often leading to overdose contributing to the opioid crisis in the United States. Therefore, understanding the structure-activity relationship between MOR and its ligands is essential for predicting MOR binding of chemicals, which could assist in the development of non-addictive or less-addictive opioid analgesics. This study aimed to develop machine learning and deep learning models for predicting MOR binding activity of chemicals. Chemicals with MOR binding activity data were first curated from public databases and the literature. Molecular descriptors of the curated chemicals were calculated using software Mold2. The chemicals were then split into training and external validation datasets. Random forest, k-nearest neighbors, support vector machine, multi-layer perceptron, and long short-term memory models were developed and evaluated using 5-fold cross-validations and external validations, resulting in Matthews correlation coefficients of 0.528–0.654 and 0.408, respectively. Furthermore, prediction confidence and applicability domain analyses highlighted their importance to the models’ applicability. Our results suggest that the developed models could be useful for identifying MOR binders, potentially aiding in the development of non-addictive or less-addictive drugs targeting MOR.

## Impact statement

This work is crucial in addressing the opioid crisis by focusing on the development of non-addictive or less-addictive opioid analgesics. Current opioids, while effective for pain relief, pose significant risks of addiction and accidental overdose. By elucidating the structure-activity relationship between the µ opioid receptor (MOR) and its ligands, this study advances the field through the development of machine learning and deep learning models to predict MOR binding activity. Evaluated via rigorous cross-validation, the models showed robust predictive capabilities. This research imparts new insights into the prediction of MOR binding, emphasizing the importance of prediction confidence and applicability domain analyses. The developed models have the potential to identify new MOR binders, significantly impacting the field by guiding the design of analgesics that mitigate the risk of addiction and overdose, ultimately improving patient safety and public health outcomes.

## Introduction

The opioid epidemic refers to the widespread misuse, addiction, and overdose deaths associated with prescription opioids and illicit drugs like heroin and synthetic opioids such as fentanyl. For many years this crisis has been a significant public health issue in the United States [1–3]. As reported by the CDC, over 105,000 drug overdose deaths were recorded in the US in 2022 [4]. Notably, between 2020 and 2021, the mortality rate from drug overdoses involving synthetic opioids excluding methadone rose by 22%, whereas deaths involving heroin decreased by 32% [5]. Until recently, the predominant cause of synthetic opioid-related deaths was attributed to fentanyl and its analogs [5, 6]. Therefore, opioid use disorder (OUD) poses a significant public health challenge, contributing to illness and mortality through addiction, overdose, and associated medical complications [7, 8]. Besides the public health issue, the opioid crisis has also caused a severe economic burden. For example, Florence et al. [9] projected the economic toll of the opioid crisis at $1.02 trillion in 2017. This encompasses the staggering costs attributed to lives lost from opioid overdose ($480.8 billion) and the diminished quality of life resulting from OUD ($390.0 billion), collectively representing more than 85% of the overall economic impact.

Numerous efforts have been dedicated to addressing the opioid crisis, encompassing enhanced regulation of opioid prescription practices [10–12], broadened accessibility to addiction treatment and harm reduction services [13–15], public awareness campaigns to highlight opioid risks [16, 17], and steps aimed at decreasing the availability of illicit opioids [9, 18]. The profound addictiveness of opioids is closely linked to the overdose fatalities caused by prescription opioids, heroin, and illicit fentanyl. However, given the important role of prescription opioids as powerful analgesics, outright prohibition of these medications is not feasible [19, 20].

Opioid drugs achieve their analgesic effects by binding to opioid receptors, including the μ opioid receptor (MOR) [21]. MOR is a primary target for analgesics. Since the discovery of MOR in the 1970s, significant efforts have been made to elucidate the relationship between the receptor and its ligands in the hopes of guiding the development of new drugs with high analgesic efficacy, fewer side effects, and a lower risk of tolerance, dependence, and addiction [22, 23]. Numerous morphine-based semi-synthetic opioids (such as oxycodone, heroin) and fully synthesized opioids (such as fentanyl) have been developed; nevertheless, none of these opioids have demonstrated both safety and efficacy as analgesics [24]. Moreover, bringing a new drug to market typically requires an investment of nearly $2.6 billion and over a decade of time [25–27]. With the increasing computational power and data sources, computational modeling using machine learning and deep learning has become a promising approach to reduce the time and cost of new drug development [21, 28–36]. Multiple computational models have been constructed for the binding activity prediction of compounds to diverse opioid receptors [37–42]. Floresta et al. [37] established three quantitative structure-activity relationship models (one field-based 3D model and two molecular fingerprint based 2D k-nearest neighbors (kNN) models) based on a dataset of 115 fentanyl-like compounds. Sakamuru et al. [38] generated models to predict both agonistic and antagonistic activity of multiple opioid receptors, including MOR, based on quantitative high-throughput screening (qHTS) assay data. Pan et al. [39] established a 3D-QSAR model to predict δ opioid receptors binding activity. The training set included 46 compounds collected from five publications. Feng et al. [40] developed machine learning and deep learning models for predicting the inhibitory activity of 75 proteins involved in opioid receptor networks, including models for MOR trained on 4,667 compounds collected from the ChEMBL database, to assess the screening and repurposing potential of more than 120,000 drug candidates targeting four opioid receptors. Leveraging transfer learning, Provasi and Filizola [41] constructed deep learning models for predicting the bioactivity of opioid receptors using ligand-based and structure-based molecular descriptors. Their MOR binding activity predictive model was trained on 87 active compounds from the IUPHAR/BPS Guide to Pharmacology database and 1,058 inactive chemicals from ChEMBL database, with inactivity determined by a -log10 of Ki, IC_50_, or EC_50_ less than 5. However, this approach raises concerns about model reliability, as many compounds defined as inactive exhibited some agonistic or antagonistic activity, increasing the potential for false negatives. Instead of predicting MOR binding activity, Oh et al. [42] developed machine learning and deep learning models for differentiating MOR agonists from antagonists. These models were trained on a small dataset (755 agonists and 228 antagonists) and evaluated with an even smaller dataset (15 agonists and 11 antagonists). The small size of the datasets and the narrow chemical space of the compounds in training these models limit the applicability of the developed models.

To enhance performance, robustness, and generalization capability of MOR binding activity prediction models, large sizes of diverse chemicals are needed in training the models. Therefore, this study collected a large size of diverse chemicals to construct machine learning and deep learning models for MOR binding activity prediction. Moreover, multiple machine learning and deep learning algorithms were adopted. We first curated MOR binding activity data of chemicals from public databases and publications. Machine learning and deep learning models were then built using multiple algorithms and validated by cross-validation and external validation. Moreover, prediction confidence and applicability domain (AD) derived from our models offer additional metrics for more appropriate applications of our models. Validation results demonstrate that the developed models could help in identifying compounds that bind to MOR, potentially facilitating the development of opioid drugs with reduced addictive properties.

## Materials and methods

### Study design

**FIGURE 1:**
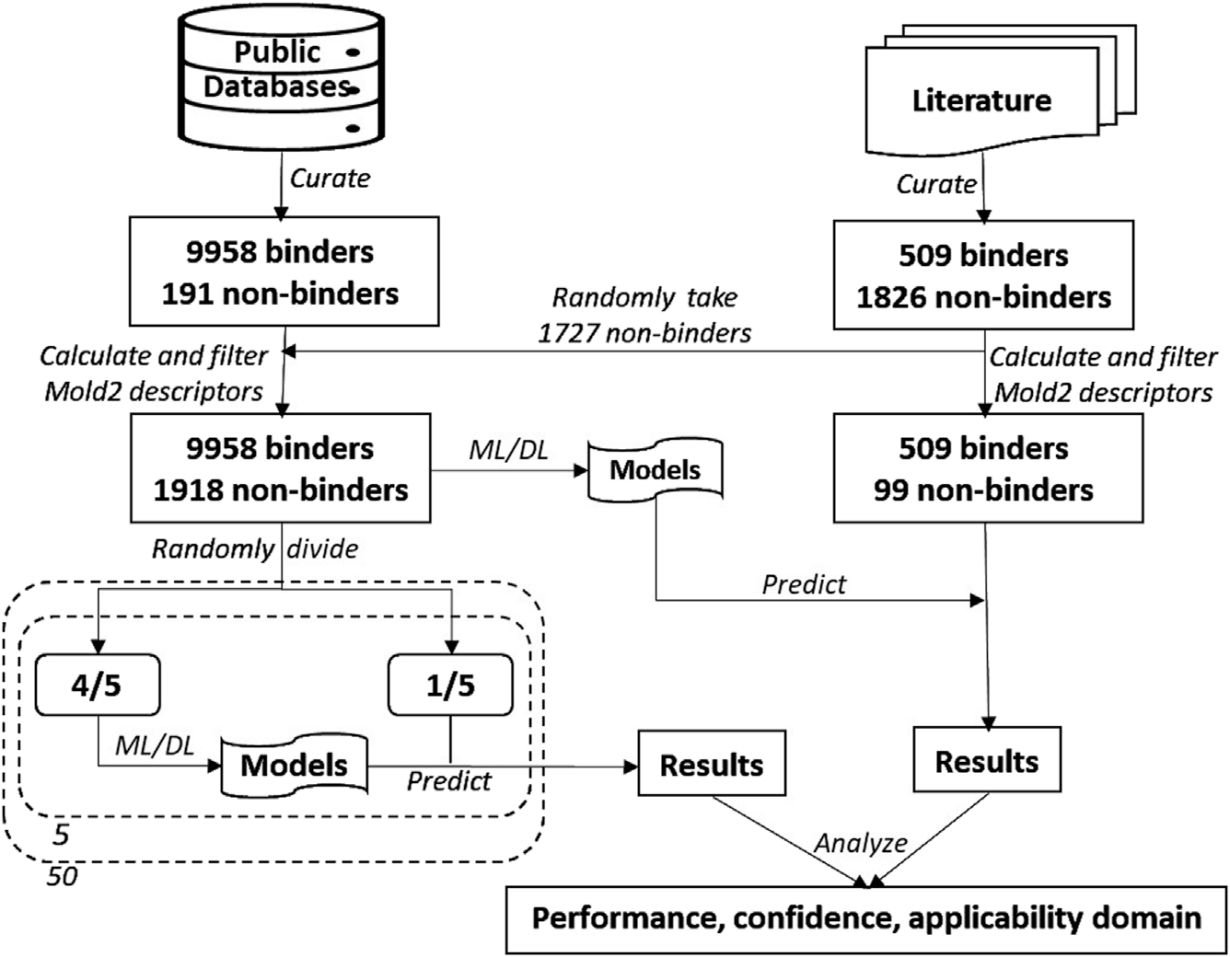
Study overview. The data on chemicals and their MOR binding activity were curated from public databases and the literature. The dataset from these databases was augmented with 1,727 non-binding chemicals sourced from the literature, forming the training dataset. The remaining chemicals from the literature constituted the external validation dataset. Molecular descriptors were calculated using Mold2 and subsequently filtered. Five algorithms—random forest, k-nearest neighbors, support vector machine, multi-layer perceptron, and long short-term memory—were used to build predictive models. The training dataset underwent 50 iterations of 5-fold cross-validation. Models constructed using the entire training dataset were then used to predict MOR binding on the external validation dataset. The performance of the models was evaluated based on their cross-validation and external validation predictions, with an additional focus on analyzing prediction confidence and applicability domain.

Study design is illustrated in Figure 1. First, chemicals with MOR binding activity data were curated from public databases as the training dataset. Chemicals with qHTS assay data reported in the literature were also curated. After removing chemicals that are contained in the training dataset, some of the inactive chemicals in qHTS assays were added to the training dataset and the rest, including active and inactive chemicals, were used as an external validation set. Molecular descriptors for the chemicals in both training and external validation datasets were then calculated using Mold2 [43, 44]. Five machine learning and deep learning algorithms, including random forest (RF) [45], kNN [46], support vector machine (SVM) [47], multi-layer perceptron (MLP) [48], and long short-term memory (LSTM) network [49], were applied in construct models. In addition, a consensus model was generated by combining the models built with each of these algorithms. Fifty iterations of 5-fold cross-validations were conducted on the training dataset for estimating the performance of the developed models. Models were constructed on the entire training dataset using these algorithms, and their generalizing capability in predicting MOR binding activity of unseen chemicals was evaluated using the external dataset. Multiple metrics were calculated for measuring model performance. At last, prediction confidence and AD were analyzed based on the predictions from both cross-validations and external validations.

### Data sources

Compounds with experimental MOR binding activity data were curated from PubChem^1^, BindingDB^2^, and ChEMBL^3^ databases. Compounds having quantitative MOR binding activity data such as IC_50_, Ki, and Kd values were designated as binders. For compounds without quantitative binding activity data, the qualitative binding activity description field was used to determine if a compound is MOR binder or non-binder. Compounds marked as “not determined” or “inconclusive” were excluded. Compounds marked as “active” or “positive” were assigned as binders, while chemicals marked as “inactive” or “negative” were treated as non-binders. Compounds with qHTS assay data on MOR used in Sakamuru et al. [38] were downloaded from^4^. The data from columns “OPRM agonist outcome” and “OPRM antagonist outcome” in both the training and validation datasets were used. A compound is inactive in both agonist and antagonist assays was termed as a non-binder, while a compound is active in one of both assays was designated as a binder. The simplified molecular input line entry system (SMILES) strings of compounds in both the public databases and the datasets from the publication were collected for representing their chemical structures.

### Data processing

The SMILES strings of the compounds obtained from the public databases (PubChem, BindingDB, and ChEMBL) and the publication (Sakamuru et al. [38]) were first converted to unique SMILES strings using the Online SMILES Translator and Structure File Generator [50]. For compounds with the same unique SMILES strings and the same activity class (binder or non-binder), only one compound was kept. Compounds with the same unique SMILES strings and different activity classes were excluded. Consequently, 10,149 compounds (9,958 binders and 191 non-binders) from the public databases and 2,527 compounds (509 binders and 2,018 non-binders) from Sakamuru et al. [38] remained. Of the 2,527 compounds, 192 non-binders are contained in the 10,149 compounds from the public databases (Supplementary Table S1) and were further removed. Finally, 509 binders and 1,826 non-binders from Sakamuru et al. were used. The binder/non-binder ratios of these two datasets (52.14 and 0.28) are dramatically different. Therefore, we randomly took 1,727 non-binders from the qHTS dataset and added them to the dataset from the public databases, resulting in a dataset of 9,958 binders and 1,918 non-binders as the training dataset (Supplementary Table S2). The remaining 509 binders and 99 non-binders from the qHTS assays were used as the external validation dataset (Supplementary Table S3). The used training and external validation datasets have similar binder/non-binder ratios. The SMILES strings of both training and external validation datasets were used to generate two-dimensional (2D) structures of the compounds using the Online SMILES Translator and Structure File Generator [50]. The SDF files obtained were used for subsequent molecular descriptors calculation.

### Descriptors calculation and filtering

Converting chemical structures into machine-readable formats is essential for developing machine learning and deep learning models [51]. In this study we utilized software tool Mold2 for calculating molecular descriptors for the compounds in the training and external validation datasets. Mold2 only accepts SDF (structure data file) representation of chemical structures [43, 44]. Therefore, the unique SMILES strings of compounds were first converted to SDF files for the training and external validation datasets using the Online SMILES Translator and Structure File Generator [50]. The generated SDF files were then input into Mold2 software for calculating molecular descriptors. Mold2 calculated 777 molecular descriptors for each compound.

Molecular descriptors with no or very low information for a dataset can significantly influence the performance of models developed using the dataset. To identify and remove such low informative descriptors, we first excluded 263 descriptors with a constant value for more than 90% of the compounds in the training dataset. Subsequently, we performed Shannon entropy analysis [43, 52–54] on the remaining 514 descriptors of the training dataset. In brief, for each molecular descriptor, the range of descriptor values of the compounds in the training dataset were first divided into 20 groups with equal value intervals. The compounds in the training dataset were then put into these 20 groups based their descriptor values. The distribution in the 20 groups, probabilities of compounds in the 20 groups, were calculated by dividing compound counts by the total compounds of the training dataset. At last, Shannon entropy values were computed for each descriptor using Equation 1.

$${H_{n}\left(p_{1},p_{2},\cdot \cdot \cdot ,p_{n}\right)=-\sum_{i=1}^{n}p_{i}\log}_{2}p_{i}$$ (1)

Where $p_{i}$ is the probability of group i . 226 descriptors with Shannon entropy less than 2.0 were considered as low informative and removed. The remaining 288 molecular descriptors have Shannon entropy values greater than or equal to 2.0 and were used in subsequent model development. The 288 molecular descriptors are listed in Supplementary Table S4. For the external validation dataset, the same 288 molecular descriptors were kept, and other descriptors were removed.

### Scaling descriptor values

The values of different molecular descriptors usually are in quite different scales in a dataset. Using unscaled molecular descriptor values to construct machine learning and deep learning models often result in low performance for most algorithms, depending on their mathematical principles. Therefore, scaling is generally needed before model development. We scaled the values of each molecular descriptor in training and external validation datasets using Equation 2.

$$V=\frac{{V_{o}-\mathrm{Min}}_{train}}{{{\mathrm{Max}}_{train}-\mathrm{Min}}_{train}}$$ (2)

Where *V* is scaled value, *V*_*o*_ is original value, *Min*_*train*_ is the minimum value of the descriptor in the training set, and *Max*_*train*_ is the maximum value in the training set.

### Model development

MOR binding activity prediction models were built using three machine learning algorithms (RF, kNN, and SVM) and two deep learning algorithms (MLP and LSTM). Numerous machine learning and deep learning algorithms have been developed, each grounded in distinct mathematical principles. kNN is a widely used, simple, and interpretable algorithm. In contrast, RF and SVM are more complex but have demonstrated good performance in various applications. However, the complexity of RF and SVM makes them challenging to interpret. We chose these three to explore the performance difference between simple and complicate machine learning models. Furthermore, MLP and LSTM represent two fundamentally different deep learning architectures: MLP is a feedforward neural network, whereas LSTM is a recurrent neural network. These two were selected to evaluate the performance of deep learning models constructed using algorithms with distinct structural designs.

When building a model using an algorithm, related algorithmic parameters were tuned through inner 5-fold cross validations. Briefly, to optimize algorithmic parameters the training set was randomly split into five folds. Four folds were used to build a model to predict the remaining fold. This process was repeated five times so that each of the five folds was used once and only once as a testing set. The prediction results on all five folds were then used to calculate a Matthews correlation coefficient (MCC) value. This inner 5-fold cross-validation was repeated five times with different random divisions of the training set into five folds. At last, the five MCC values from five iterations of inner 5-fold cross-validations were averaged to estimate performance of models built with a set of parameters. The set of hyperparameters resulting in the highest average MCC value was determined as the optimized parameters for the algorithm and were used to construct a model on the training set.

The hyperparameters tuned in our study are given below. For RF, n_estimators (100 and 200 trees), min_samples_leaf (10 and 20), and max_chemical (1,000 and 2,000) were tuned. For kNN, the parameters n_neighbors (k = 3, 5, and 7) and weights (“uniform,” “distance”) were optimized. For SVM, a linear kernel was employed and the regularization parameter C (0.1, 1, and 10) was optimized. For MLP, alpha (0.0001, 0.1) and hidden_layer_sizes (100, 300) were optimized. For LSTM, the number of epochs was tuned to 500 with running 5,000 epochs based on the training loss value and accuracy. Other parameters used for LSTM include recurrent layers = 4, features = 200, batch size = 32, and learning rate = 0.0001. For these five algorithms, except the parameters aforementioned, default values were adopted for other algorithmic parameters.

The RF, kNN, SVM, and MLP models were constructed using the packages in Scikit-learn (0.23.2) [55] in Python (3.8.5) [56], while the LSTM models were developed using a PyTorch package (2.0.1) [57] in Python (3.8.5).

In addition to models developed using the five algorithms, a consensus model was constructed for a training set using the five individual models. Each of these models was built using distinct algorithms, potentially utilizing different features from the same training dataset. Consensus modeling capitalizes on the strengths of each model, aggregating their predictions to deliver more reliable, robust, and accurate results. This approach enhances the overall performance by minimizing the weaknesses inherent in any single model. The consensus model combines outcomes from its five individual models using a majority voting strategy: if three or more individual models predict a compound as MOR binder, the compound is determined as a binder, otherwise, it is predicted as non-binder.

### Model evaluation

Model performance was evaluated using two strategies: 5-fold cross-validation and external validation. In a 5-fold cross-validation, the entire training set was first randomly divided into five equal or close to equal folds. Four of the five folds were then used to tune algorithmic parameters using the inner 5-fold cross-validations for each of the machine learning and deep learning algorithms. The tuned parameters were then used to train models on the four folds, and the trained models were used to predict the remaining fold. This process was repeated five times so that each of the five folds was used as a testing set only once. At last, performance metrics values were calculated using prediction results from all five testing sets to estimate model performance. The 5-fold cross-validation was repeated 50 times to reach statistically robust estimations on model performance.

The training dataset was randomly split into five subsets, four subsets for training and one subset for testing. This random splitting was repeated five times to ensure all compounds were used for both training and testing.

External validation was employed to evaluate the generalization of the constructed models using the entire training set. The same parameter tuning process was applied to the whole training set. The optimized parameters were then used to develop models using the entire training set. Finally, the developed models were used to predict MOR binding activity for compounds in the testing set.

### Performance metrics

Five metrics were used to measure model performance, including accuracy, sensitivity, specificity, balanced accuracy, and MCC. These metrics were derived by comparing model predictions with actual binding activity data. They were calculated using Equations 3–7.

$$\text{Accuracy}=\frac{TP+TN}{TP+TN+FP+FN}$$ (3)

$$\text{Sensitivity}=\frac{TP}{TP+FN}$$ (4)

$$\text{Specificity}=\frac{TN}{TN+FP}$$ (5)

$$\text{Balanced Accuracy}=\frac{\text{Sensitivity}+\text{Specificity}}{2}$$ (6)

$$\text{MCC}=\frac{\text{TP}*\text{TN}-\text{FP}*\text{FN}}{\sqrt{\left(TP+FP\right)*\left(TP+FN\right)*\left(TN+FP\right)*\left(TN+FN\right)}}$$ (7)

Where TP, TN, FP, and FN represent true positives, true negatives, false positives, and false negatives, respectively.

### Prediction confidence analysis

Predictions produced by our machine learning or deep learning models provide not only class assignments but also probabilities that quantify the likelihoods of these class assignments. The prediction probability of a prediction not only classifies the compounds as MOR binder or non-binder, but also measures the confidence of the prediction. Prediction confidence analysis is conducted to evaluate if prediction confidence can be used as an additional valuable parameter to inform better utilization of a model in applications, such as decision-making and safety assessment. The prediction confidence of a prediction is derived from the prediction probability using Equation 8 [43, 53, 54, 58].

$$\text{Prediction confidence}=\frac{\left|\text{prob}-0.5\right|}{0.5}$$ (8)

where *prob* is the probability of a compound predicted as a MOR binder from a machine learning and deep learning model. Prediction confidence values are between 0 and 1. The larger the value the more confidence in the prediction.

To examine the relationship between prediction confidence and prediction performance for predictions of a model in 5-fold cross-validations or external validation, the prediction confidence value range (between 0 and 1) was divided into 10 even bins with the interval of 0.1. Next, the predictions were allocated to the 10 bins according to their prediction confidence values. Lastly, performance metrics were separately calculated for predictions in each the 10 bins.

### Applicability domain (AD) analysis

AD of a model represents the structural space of chemicals utilized to train the model. Chemicals falling within the AD of a model exhibit structural similarities to the training chemicals, thus yielding more accurate predictions. Therefore, AD analysis plays a crucial role in evaluating the predictions made by computational models [59–61]. In this study, the AD of a model was defined by the boundaries of all descriptors ranging from the minimum to the maximum values of chemicals used in training the model. More specifically, we first computed the AD of a model using the training chemicals. Next, the distance of a chemical to the AD was calculated using Equation 9.

$$Distance=\sqrt{{d_{1}}^{2}+{{{d_{2}}^{2}+\ldots +d}_{n}}^{2}}$$ (9)

Where *d*_*i*_ (i = 1, 2, …, n) is the distance of the chemical to the AD for molecular descriptor *i*. If the value of molecular descriptor *i* falling in the value range of the same molecular descriptors of the training chemicals, d_i_ was set to zero. Therefore, when all molecular descriptor values of a chemical fall within the molecular descriptor value ranges of training chemicals, the *Distance* value is calculated to be zero according to Equation 9 and the chemical is considered inside the model’s AD. If the value of any molecular descriptor is outside the descriptor value boundary of the training set, the *Distance* value is greater than zero and the chemical is considered outside the model’s AD. At last, performance of predictions inside and outside AD was compared for the models constructed in the 5-fold cross-validations and the model built in the external validation.

## Results

### Model performance

**FIGURE 2:**
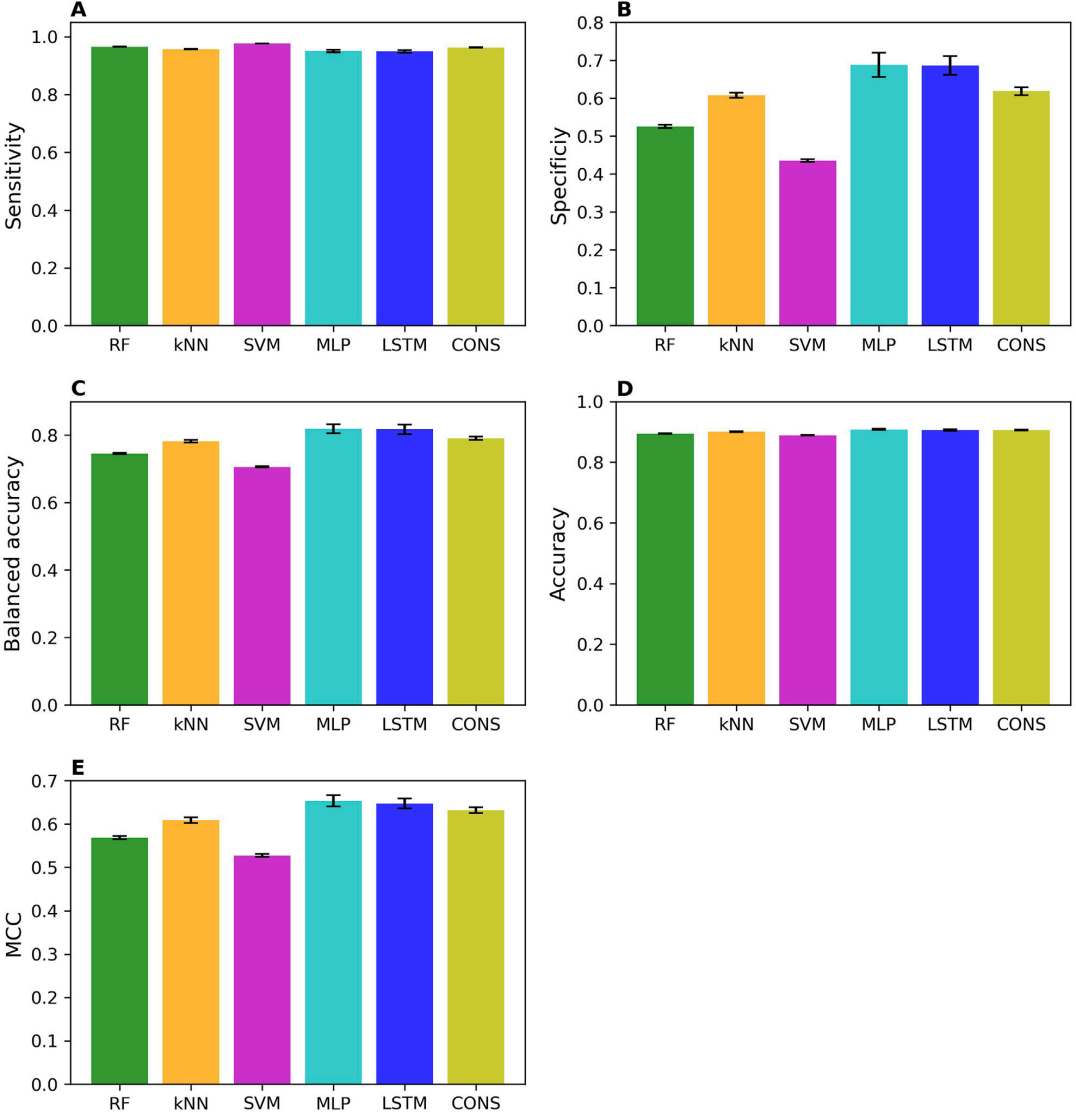
Performance of cross-validations. Performance of 50 iterations of 5-fold cross-validations was measured using sensitivity **(A)**, specificity **(B)**, balanced accuracy **(C)**, accuracy **(D)**, and MCC **(E)**. The average values of these metrics across the 50 iterations are represented by color bars, corresponding to different algorithms indicated by the x-axis labels (RF, random forest; kNN, k-nearest neighbors; SVM, support vector machine; MLP, multi-layer perceptron; LSTM, long short-term memory; and CONS, consensus model). The standard deviations are displayed as error bars on top of the color bars.

The prediction performances of the machine learning (RF, kNN, and SVM), deep learning (MLP and LSTM), and consensus models from the 50 iterations of 5-fold cross-validations were summarized in Figure 2 in sensitivity (Figure 2A), specificity (Figure 2B), balanced accuracy (Figure 2C), accuracy (Figure 2D), and MCC (Figure 2E). Overall, all models performed well, as indicated by the averaged performance metrics values (the bars in Figure 2). More specifically, performance metrics accuracy (0.89 – 0.91), balanced accuracy (0.71 – 0.82), and MCC (0.53 – 0.65) are high for all models, indicating good overall performance. Not surprisingly, all models performed much better on MOR binders than non-binders, with much higher averaged sensitivity (0.95 – 0.98) than specificity (0.44 – 0.69) because the training dataset has a greater number of MOR binders (9,958) than non-binders (1,918). Notably, all performance metrics exhibit small standard deviations among the 50 iterations of cross-validations (the sticks atop the bars in Figure 2), suggesting that the machine learning, deep learning, and consensus models were relatively unaffected by the random partitioning of the whole training dataset into five folds. Interestingly, the two deep learning models (the cyan and blue bars in Figure 2) outperformed the three machine learning models (the green, light brown, and magenta bars in Figure 2), especially in specificity (Figure 2B). These results demonstrate that a large dataset, such as the one training dataset in this study with 11,876 chemicals, is needed for deep learning algorithms to show superiority over conventional machine learning algorithms, especially for the minority class of an imbalanced dataset. Surprisingly, the consensus models did not surpass all member models. They performed better than the three machine learning models but worse than the two deep learning models. Our results indicate that though consensus modeling remains as an effective approach to combine models constructed using conventional machine learning algorithms, it deserves further investigation on if and how a consensus approach can be applied to models built with deep learning algorithms as we only used one consensus strategy, majority voting.

**FIGURE 3:**
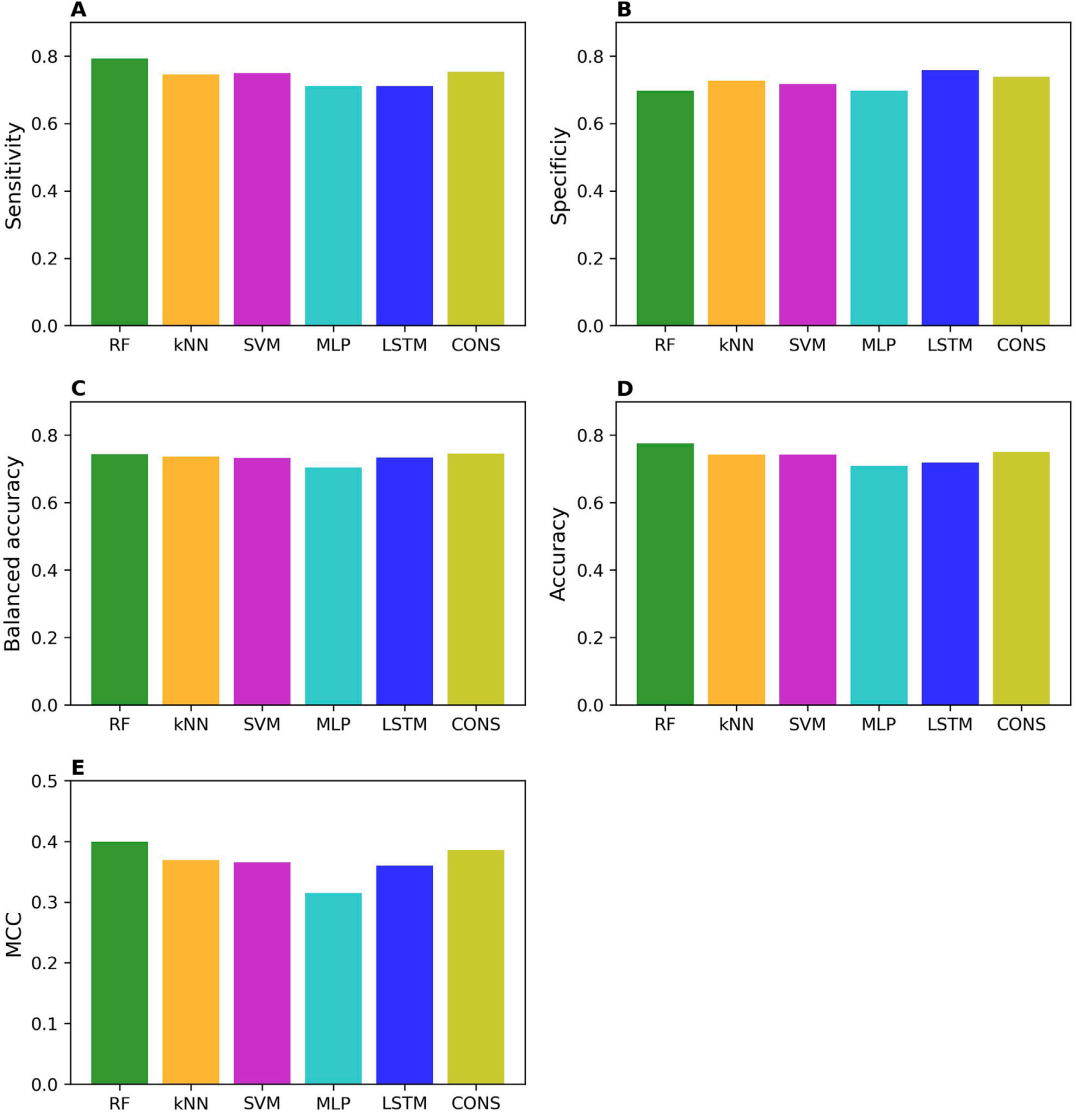
Performance of external validations. The performance was assessed using sensitivity **(A)**, specificity **(B)**, balanced accuracy **(C)**, accuracy **(D)**, and MCC **(E)**. The values of these metrics are represented by color bars for models developed using different algorithms, as indicated by the x-axis labels (RF, random forest; kNN, k-nearest neighbors; SVM, support vector machine; MLP, multi-layer perceptron; LSTM, long short-term memory, and CONS, consensus model).

The external validation performances on the models trained with the whole training set are summarized in Figure 3. The external validation results indicate that the models performed well, with good performance metrics values; sensitivity of 0.71–0.79, specificity of 0.70–0.76, balanced accuracy of 0.70–0.74, accuracy of 0.71–0.78, and MCC of 0.32–0.40. As expected, they slightly underperformed the models in the cross-validations. Strikingly, specificity and sensitivity are very close in the external validations, in contrast to the cross-validation results where sensitivity is expectedly higher than specificity. This difference may attribute to the nature of data in the training and external validation datasets. The MOR binders in the training dataset are determined by conventional low-put assays, resulting in models that perform well in predicting chemicals tested with the same assays. In contrast, most of the MOR non-binders in the training dataset are results from qHTS assays, and thus the trained models performed better on the external MOR non-binders determined by the same qHTS assays. Our results suggest that caution should be exercised in the validation and application of machine learning and deep learning models.

Surprisingly, not like in the cross-validations, the two deep learning models did not consistently outperform all three machine learning models in the external validations.

### Prediction confidence analysis

**FIGURE 4:**
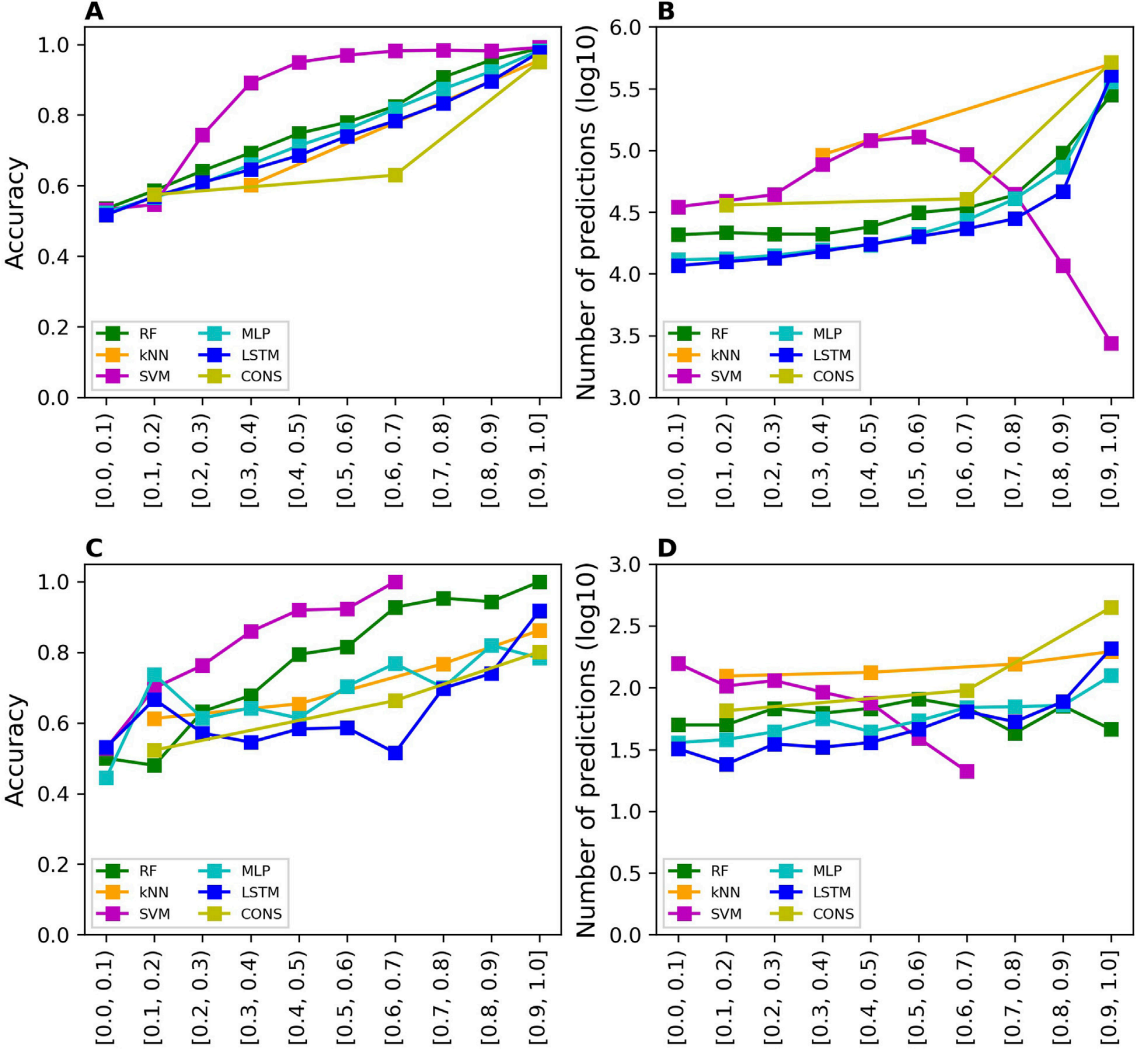
Prediction confidence analysis results. The analysis of prediction confidence is depicted by plotting prediction accuracy values and the number of predictions at various confidence levels. **(A, B)** show the results for cross-validations, while **(C, D)** display the results for external validations. The x-axis tick labels represent the different confidence levels. The models developed using different algorithms are distinguished by various colors, as indicated in the color legend (RF, random forest; kNN, k-nearest neighbors; SVM, support vector machine; MLP, multi-layer perceptron; LSTM, long short-term memory, and CONS, consensus model).

The prediction confidence analysis was performed on the results of cross-validations and external validation. The accuracy values of the predictions at 10 confidence levels from the cross-validations are shown in Figure 4A for all models. The accuracy of predictions is improved when their prediction confidence level is increased, for all models. Similar trends were observed for sensitivity, specificity, balanced accuracy, and MCC as depicted in Supplementary Figures S1–S4, respectively. Moreover, more predictions fall in higher confidence levels for all models except SVM as shown in Figure 4B.

The prediction confidence analysis was also conducted on the results of external validation. The accuracy values of the predictions at 10 confidence levels from the external validations are shown in Figure 4C for all models. The trends are similar to those observed in the cross-validations: higher prediction confidence levels correspond to greater prediction accuracy. However, the trend lines are less smooth than those in the cross-validations, due to the significantly fewer predictions at each confidence level. The sensitivity, specificity, balanced accuracy, and MCC values of the predictions at 10 confidence levels from the external validations exhibit similar trends as shown in Supplementary Figures S5–S8, respectively. Notably, the SVM model had very few predictions at high confidence levels, 3, 1, and 3 at confidence levels 0.7–0.8, 0.8–0.9, and 0.9–1.0, respectively. Therefore, performance metrics at these confidence levels were not calculated because they would not be statistically meaningful. The number of predictions is plotted against prediction confidence level in Figure 4D. In general, the number of predictions does not differ much in confidence levels except the SVM model which had fewer predictions at higher confidence levels.

### Applicability domain analysis

**FIGURE 5:**
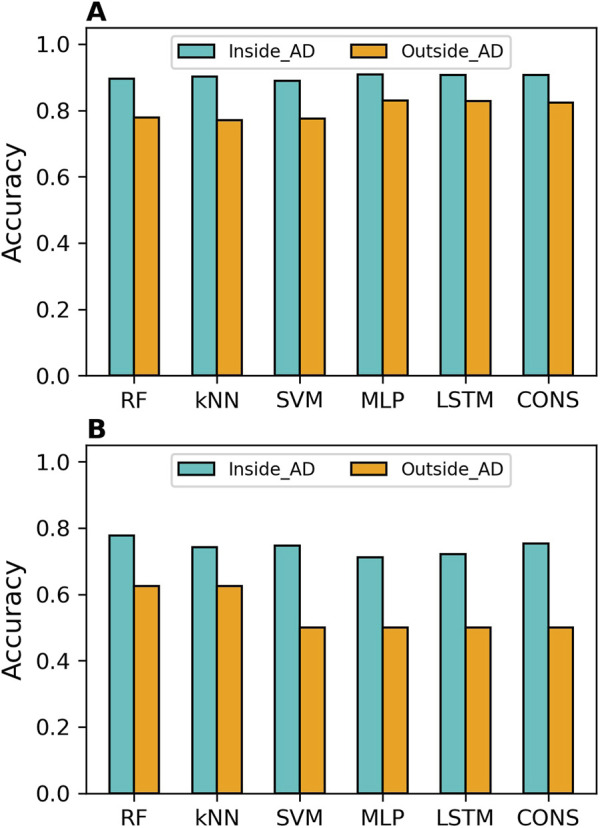
Applicability domain (AD) analysis results. The AD analysis is presented for cross-validations **(A)** and external validations **(B)**. Accuracy for predictions within the AD is shown in cyan bars, while accuracy for predictions outside the AD is displayed in orange bars. The models developed using different algorithms are represented by the x-axis labels (RF, random forest; kNN, k-nearest neighbors; SVM, support vector machine; MLP, multi-layer perceptron; LSTM, long short-term memory, and CONS, consensus model).

The distances to the AD of the compounds predicted in the cross-validations and external validations were computed. Prediction accuracy values inside and outside the AD were calculated separately and are illustrated in Figure 5A for the cross-validations and Figure 5B for the external validation. It was clear that the compounds inside the AD were predicted more accurately than those outside the AD by all models, in both cross-validations and external validations. The sensitivity, specificity, balanced accuracy, and MCC values of predictions inside and outside AD for all models are presented in Supplementary Figures S9–S12, respectively, for the cross-validations, and in Supplementary Figures S13–S16 for the external validations.

In terms of overall performance metrics (accuracy, balanced accuracy, and MCC), all models performed better inside the AD than outside it. However, examining performance on binders and non-binders, it was found that the compounds outside the AD had higher specificity than those inside the AD for all models in both cross-validations and external validations. This indicates that all models performed better on MOR binders than on non-binders. Moreover, the deep learning models achieved higher MCC and balanced accuracy than the machine learning models, both inside and outside the AD, in both cross-validations and external validation.

The results demonstrated that AD analysis is beneficial for evaluating the reliability of predictions from both machine learning and deep learning models. It is worth noting that the MCC values for predictions outside the AD in the external validations are zeros for SVM, MLP, and LSTM models (Supplementary Figure S16). This might not be statistically robust due to the small number (8) of compounds.

## Discussion

The opioid epidemic is a severe public health crisis in the United States, leading to an increasing number of deaths and imposing a substantial economic burden. Opioids are potent analgesics, but many are addictive and prone to cause an overdose. Hence, the non- or less-addictive drugs that target MOR are needed. Developing new drugs is a lengthy and costly process, often taking about a decade and billions of dollars. Therefore, computational approaches provide a promising and efficient way to aid drug development. In this study, we collected chemicals with MOR binding activity data from multiple databases and the literature. We then constructed and evaluated machine learning and deep learning models using the curated data for MOR binding activity prediction.

The curated data are imbalanced, which is common in the real world. The data collected from databases have a greater number of MOR binders than non-binders. Conversely, the qHTS data acquired from the literature have more MOR non-binders than binders. Hence, to maintain a consistent ratio of binders to non-binders in both the training and testing datasets, 1,727 non-binders were randomly taken from the qHTS data and added to the data collected from databases to form the training dataset. The remaining qHTS data were used as the external validation dataset. The same prevalence of MOR binders in both the training and external validation datasets can reduce the impact of difference in prevalence on external validation results, enhancing the reliability of extrapolation assessment for the developed models.

Both the training and external validation datasets are biased toward MOR binders. In two-class classification models, accuracy tends to favor the majority class, which, in this study, is the MOR binders. This bias affects the evaluation of prediction performance, especially in imbalanced datasets. Therefore, we also employed balance accuracy and MCC to measure overall performance.

Interestingly, all models had higher sensitivity than specificity, especially in the cross-validations (Figures 2A, B). This discrepancy arises because the training dataset contains a greater number of MOR binders than non-binders, enabling the models to learn the structures of MOR binders better than those of non-binders. Consequently, this results in more accurate predictions for binders (higher sensitivity) compared to non-binders (lower specificity). These findings suggest that incorporating more non-binders into the training dataset would likely improve the prediction performance of machine learning and deep learning models. To address this issue, future efforts should focus on incorporating a more balanced representation of non-binders in the training dataset. This would help reduce the imbalance and, in turn, enhance the robustness and reliability of the models. Additionally, we recommend that the scientific community place equal value on the publication of inactive results, alongside active findings. Acknowledging and appreciating inactive data will not only contribute to a more balanced dataset but also foster greater transparency and scientific rigor in the field.

It is worth noting that the prediction performance of all models in the external validations is worse than in the cross-validations. This discrepancy is not surprising because the MOR binding activity data in the training and external validation datasets are obtained from different types of assays. The training dataset, except for 1,727 non-binders, consists of results from traditional assays, whereas the external validation dataset is generated using the qHTS assay. Hence, the poorer performance of the machine learning and deep learning models in the external validations compared to the cross-validations cannot be fully attribute to the models extrapolating to different chemicals. The difference in experimental methods that produced the MOR binding activity data in the two datasets likely contribute to this performance discrepancy.

To confirm this hypothesis, we examined the concordance between the two types of data. There are 192 compounds in the training dataset with qHTS assay data that were excluded from the external validation dataset. Of these 192 compounds, 186 are binders and only 6 are non-binders according to traditional assays, while the qHTS assay results show 42 binders and 150 non-binders. Comparison revealed that 41 binders and five non-binders are common in the two methods. Hence, most binders from the qHTS assay (41 out of 42) can be predicted using traditional assay results, whereas only a few non-binders (5 out of 150) can be predicted. This low concordance between the qHTS assay data and traditional assay data confirms our hypothesis.

Various consensus strategies can be employed to combine multiple individual models into a unified consensus model. In this study, consensus models were generated using a majority voting strategy based on the predictions of five individual models. To further investigate the potential impact of different consensus strategies, we also applied an average prediction probability approach. In this method, the mean of the MOR binder prediction probabilities from the five individual models was calculated and used as the binder probability for the consensus model. If the consensus probability of a compound was greater than or equal to 0.5, it was predicted as a binder; otherwise, it was classified as a non-binder. The consensus models derived from both the majority voting and average prediction probability strategies exhibited similar performance, as shown in Supplementary Figure S17.

A machine learning and deep learning model not only predicts the classes of a sample but also quantifies the likelihood of the sample belonging to the predicted class. In this study, the models output a probability indicating the likelihood of a compound being a binder. This probability is used not only to predict the compound as a MOR binder or non-binder, but also to measure the confidence of the prediction. To evaluate the usefulness of this probability, prediction confidence analysis was conducted on predictions in both cross-validations and external validations. The results (Figure 4) suggest that prediction confidence derived from the developed models offers an additional valuable metric for their applications.

The interpretability of deep learning models remains a key challenge, particularly in complex domains such as predicting binding activity for MOR. While the two deep learning models achieved higher predictive performance than the three machine learning models, the black-box nature makes deep learning models difficult to directly understand how individual molecular descriptors influence MOR binding. To enhance interpretability, techniques such as feature importance analysis, SHAP (Shapley Additive Explanations), and LIME (Local Interpretable Model-agnostic Explanations) can be used to identify the most influential molecular descriptors. However, achieving a fully transparent understanding of deep learning model behavior remains an ongoing research challenge. In the context of this study, we focus on predictive accuracy but acknowledge the need for further work on improving model interpretability for better insight into the underlying mechanisms of MOR binding.

AD serves as a critical metric for evaluating the uncertainty of predictions from machine learning or deep learning models. Compounds within the chemical space of the training compounds, or within the AD of a model, are expected to be predicted more accurately than those outside the AD [60, 61]. Our AD analysis of the predictions in the 5-fold cross-validations (Figure 5A) and external validations (Figure 5B) revealed that predictions within the AD are more accurate than those outside the AD for all models. Therefore, developing a model based on a training dataset with a broader chemical space can improve its applicability to a wider range of chemicals.

The scope of application of a predictive model is determined by its training dataset. The models developed in this study are based on a large dataset derived from traditional low-throughput experiments. In these models, any compound that exhibits binding activity—regardless of how weak—is classified as a MOR binder. As a result, the cross-validation results reflect the accuracy of predictions for chemicals in these binding assays, while the external validation results assess the models’ ability to generalize and predict the binding activity of compounds in qHTS assays. To improve the reliability of predictions for MOR binding activity in either traditional or qHTS assays, stratified sampling should be employed to generate training and testing datasets. To demonstrate this, we applied this strategy. The details of the process are provided in the Supplementary Material, and the results are summarized in Supplementary Figures S18–S19.

In conclusion, machine learning (RF, kNN, and SVM) and deep learning (MLP and LSTM) models were constructed for MOR binding activity prediction. These models were evaluated using 5-fold cross-validations and external validations. The models achieved good performance in both evaluation methods. Results from prediction confidence analysis and AD analysis demonstrated the importance of prediction confidence and AD in evaluating the reliability of the models’ predictions. Our findings suggest that the developed models have the potential to identify MOR binders, which could assist in the development of non-addictive or less-addictive drugs targeting MOR.

## Data Availability

Publicly available datasets were analyzed in this study. This data can be found in the article/Supplementary Material.

## Footnotes

1
https://pubchem.ncbi.nlm.nih.gov/

2
https://www.bindingdb.org/rwd/bind/index.jsp

3
https://www.ebi.ac.uk/chembl/

4
https://tripod.nih.gov/tox/oprpred/codes.zip
