# Supplementary material for: Developing predictive models for µ opioid receptor binding using machine learning and deep learning techniques
Source: Exp Biol Med (Maywood). 2025 Mar 19;250:10359. doi: 10.3389/ebm.2025.10359 (PMC11961360; doi:10.3389/ebm.2025.10359)
Supplement: Supplementary file 1 [file DataSheet6.docx]

Developing Predictive Models for µ Opioid Receptor Binding Using Machine Learning and Deep Learning Techniques

Jie Liu^1^, Jerry Li^2^, Zoe Li^1^, Fan Dong^1^, Wenjing Guo^1^, Weigong Ge^1^, Tucker A. Patterson^1^, Huixiao Hong^1*^

^1^National Center for Toxicological Research, U.S. Food & Drug Administration, 3900 NCTR Road, Jefferson, AR 72079

^2^ Department of Computer Science, Rice University, 6100 Main Street, Houston, TX 77005

^*^Corresponding author: Dr. Huixiao Hong, NCTR/FDA, 3900 NCTR Road, Jefferson, AR 72079. E-mail: huixiao.hong@fda.hhs.gov

**Running title:**  Predictive Models for µ Opioid Receptor Binding

**Supplementary Figures**

**Figure S1**. The sensitivity of prediction confidence analysis from cross-validations 3

**Figure S2**. The specificity of prediction confidence analysis from cross-validations 4

**Figure S3**. The balanced accuracy of prediction confidence analysis from cross-validations 5

**Figure S4**. The MCC of prediction confidence analysis from cross-validations 6

**Figure S5**. The sensitivity of prediction confidence analysis from external validation 7

**Figure S6**. The specificity of prediction confidence analysis from external validation 8

**Figure S7**. The balanced accuracy of prediction confidence analysis from external validation 9

**Figure S8**. The MCC of prediction confidence analysis from external validation 10

**Figure S9**. The sensitivity of applicability domain analysis of cross-validations 11

**Figure S10**. The specificity of applicability domain analysis of cross-validations 12

**Figure S11**. The balanced accuracy of applicability domain analysis of cross-validations 13

**Figure S12**. The MCC of applicability domain analysis of cross-validations 14

**Figure S13**. The sensitivity of applicability domain analysis of external validation 15

**Figure S14**. The specificity of applicability domain analysis of external validation 16

**Figure S15**. The balanced accuracy of applicability domain analysis of external validation 17

**Figure S16**. The MCC of applicability domain analysis of external validation 18

**Figure S17**. The performance of consensus models 19

**Figure S18**. The performance of cross-validations from stratified selected data 20

**Figure S19**. The performance of external validation from stratified selected data 21


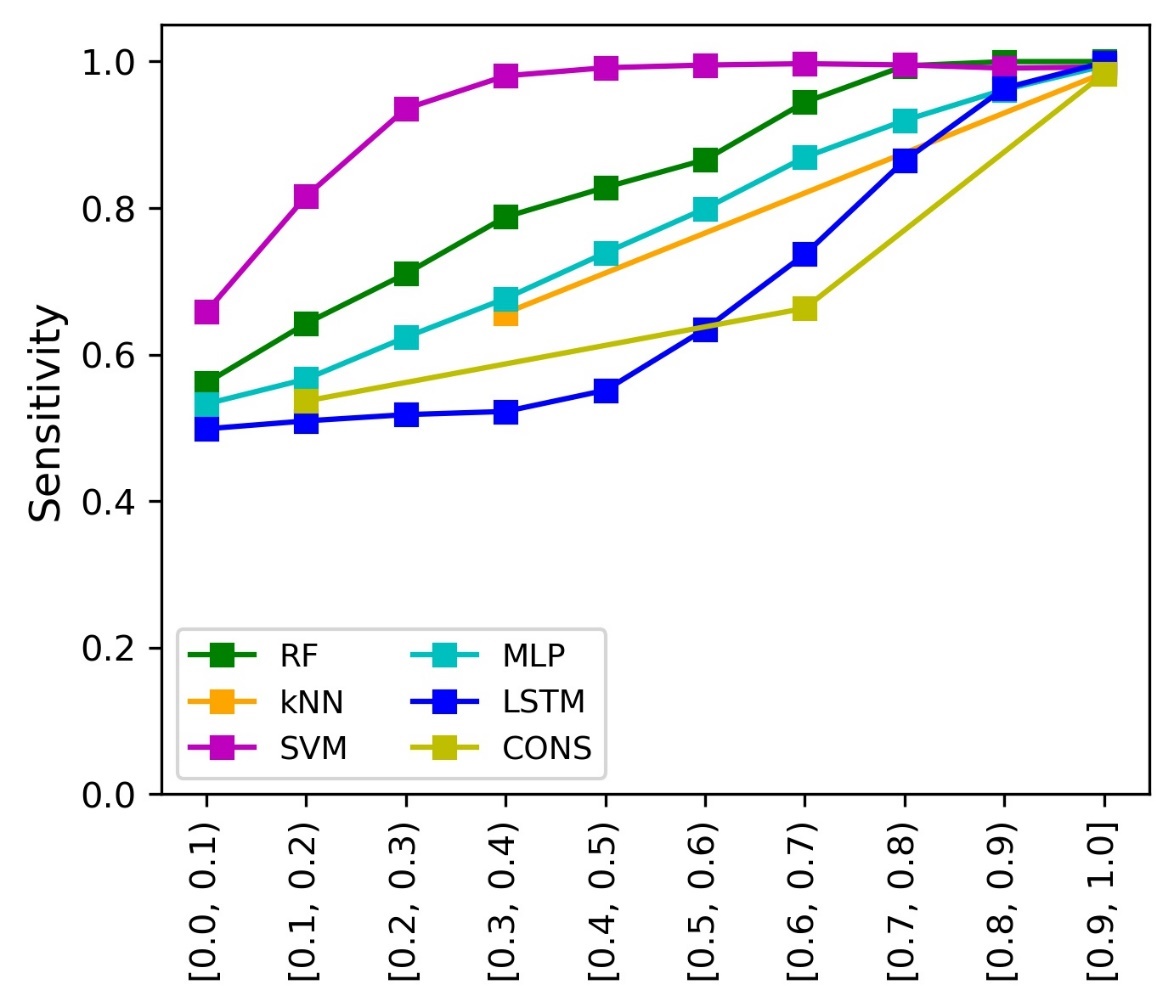


**Figure S1.** The sensitivity of prediction confidence analysis from cross-validations. The x-axes indicate the ten prediction confidence levels. The sensitivity values were plotted as the green, orange, magenta, cyan, blue, and yellow lines for Random Forest (RF), k-nearest neighbors (kNN), support vector machine (SVM), Multi-layer Perceptron Classifier (MLP), Long Short-Term Memory (LSTM), and consensus model (CONS), respectively.

**
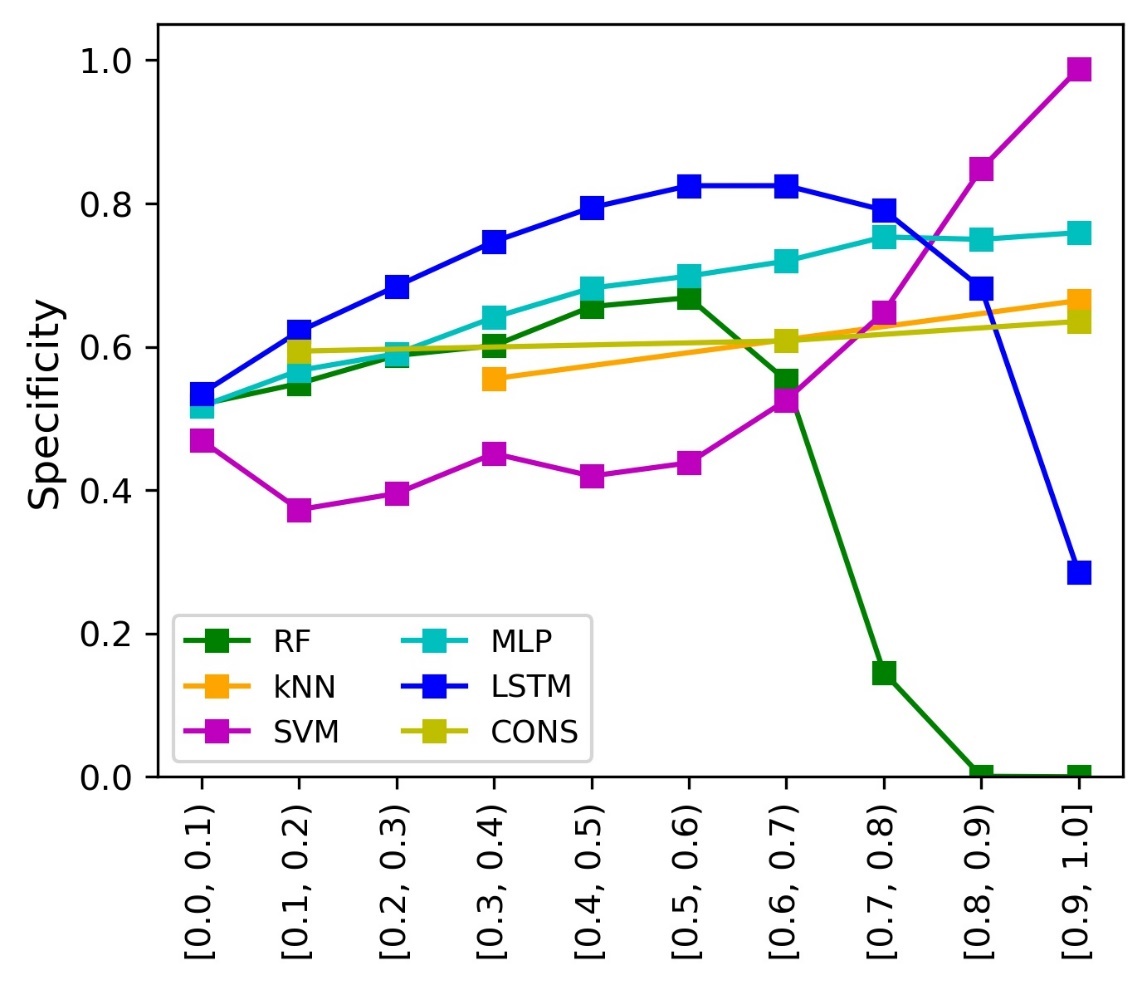
**

**Figure S2.** The specificity of prediction confidence analysis from cross-validations. The x-axes indicate the ten prediction confidence levels. The specificity values were plotted as the green, orange, magenta, cyan, blue, and yellow lines for Random Forest (RF), k-nearest neighbors (kNN), support vector machine (SVM), Multi-layer Perceptron Classifier (MLP), Long Short-Term Memory (LSTM), and consensus model (CONS), respectively.

**
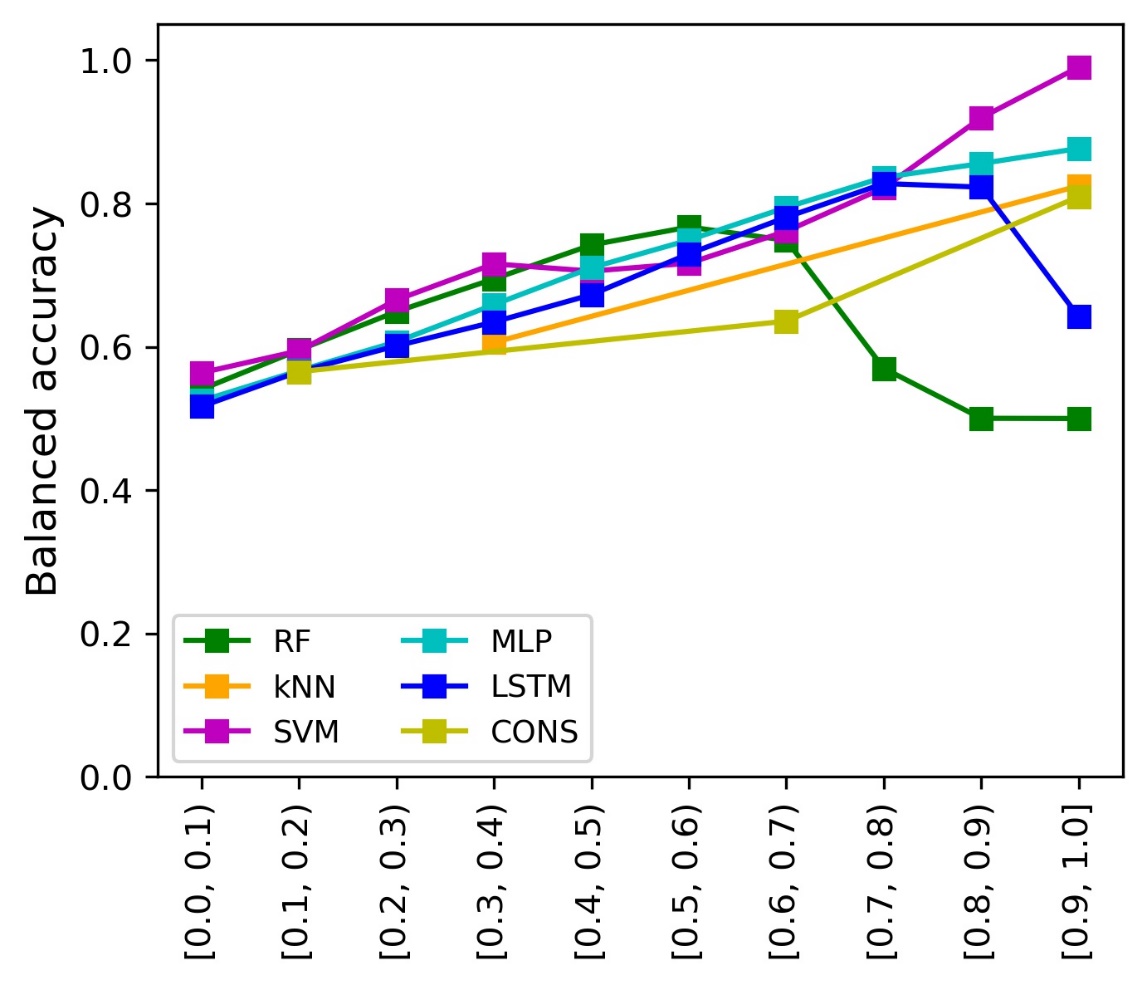
**

**Figure S3.** The balanced accuracy of prediction confidence analysis from cross-validations. The x-axes indicate the ten prediction confidence levels. The balanced accuracy values were plotted as the green, orange, magenta, cyan, blue, and yellow lines for Random Forest (RF), k-nearest neighbors (kNN), support vector machine (SVM), Multi-layer Perceptron Classifier (MLP), Long Short-Term Memory (LSTM), and consensus model (CONS), respectively.

**
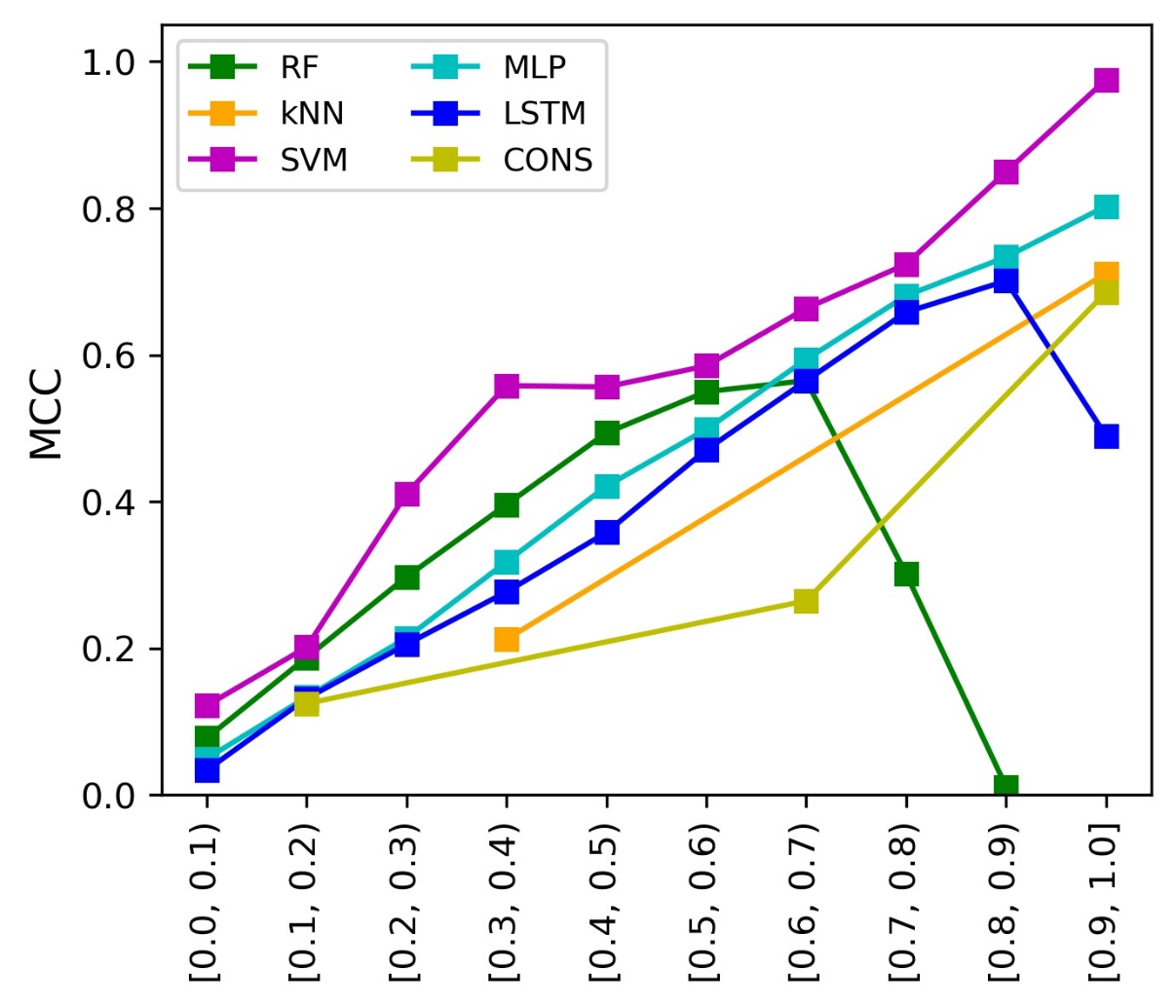
**

**Figure S4.** The MCC of prediction confidence analysis from cross-validations. The x-axes indicate the ten prediction confidence levels. The MCC values were plotted as the green, orange, magenta, cyan, blue, and yellow lines for Random Forest (RF), k-nearest neighbors (kNN), support vector machine (SVM), Multi-layer Perceptron Classifier (MLP), Long Short-Term Memory (LSTM), and consensus model (CONS), respectively.

**
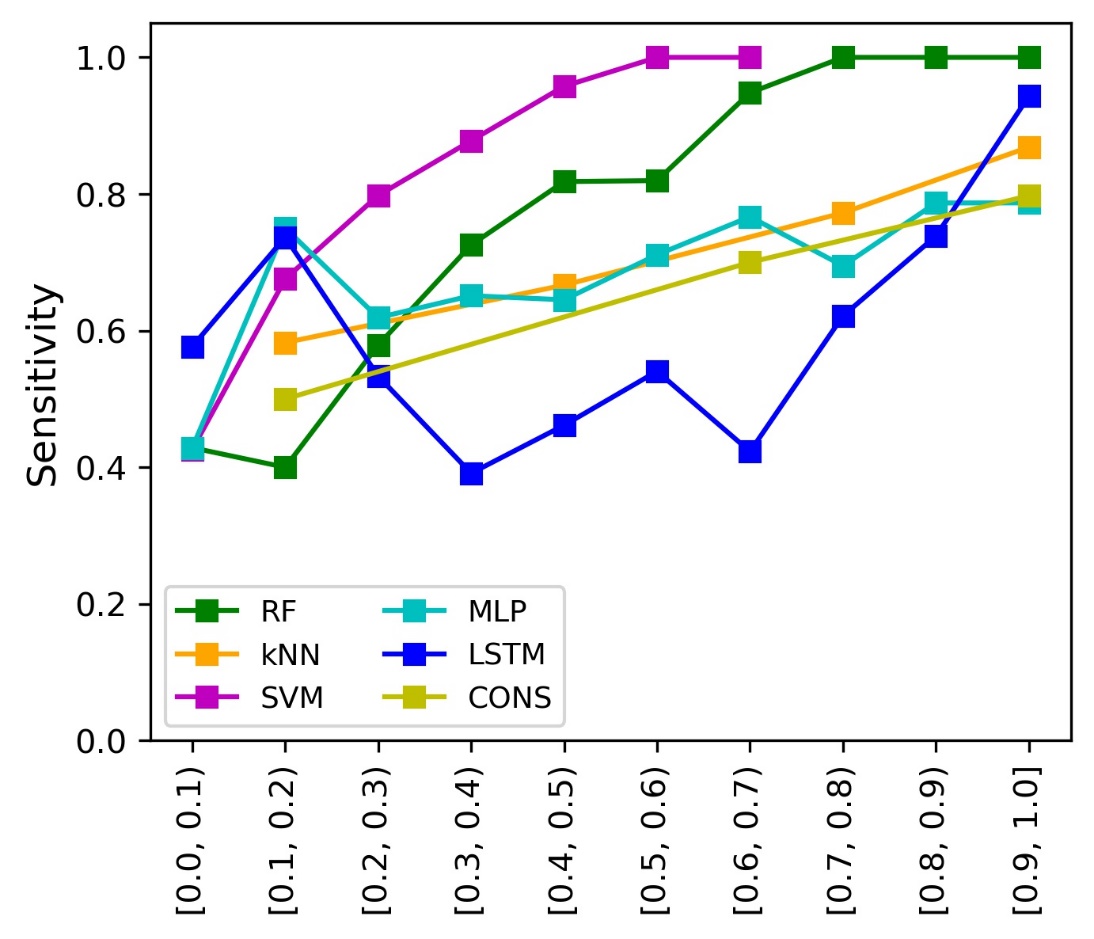
**

**Figure S5.** The sensitivity of prediction confidence analysis from external validation. The x-axes indicate the ten prediction confidence levels. The sensitivity values were plotted as the green, orange, magenta, cyan, blue, and yellow lines for Random Forest (RF), k-nearest neighbors (kNN), support vector machine (SVM), Multi-layer Perceptron Classifier (MLP), Long Short-Term Memory (LSTM), and consensus model (CONS), respectively.

**
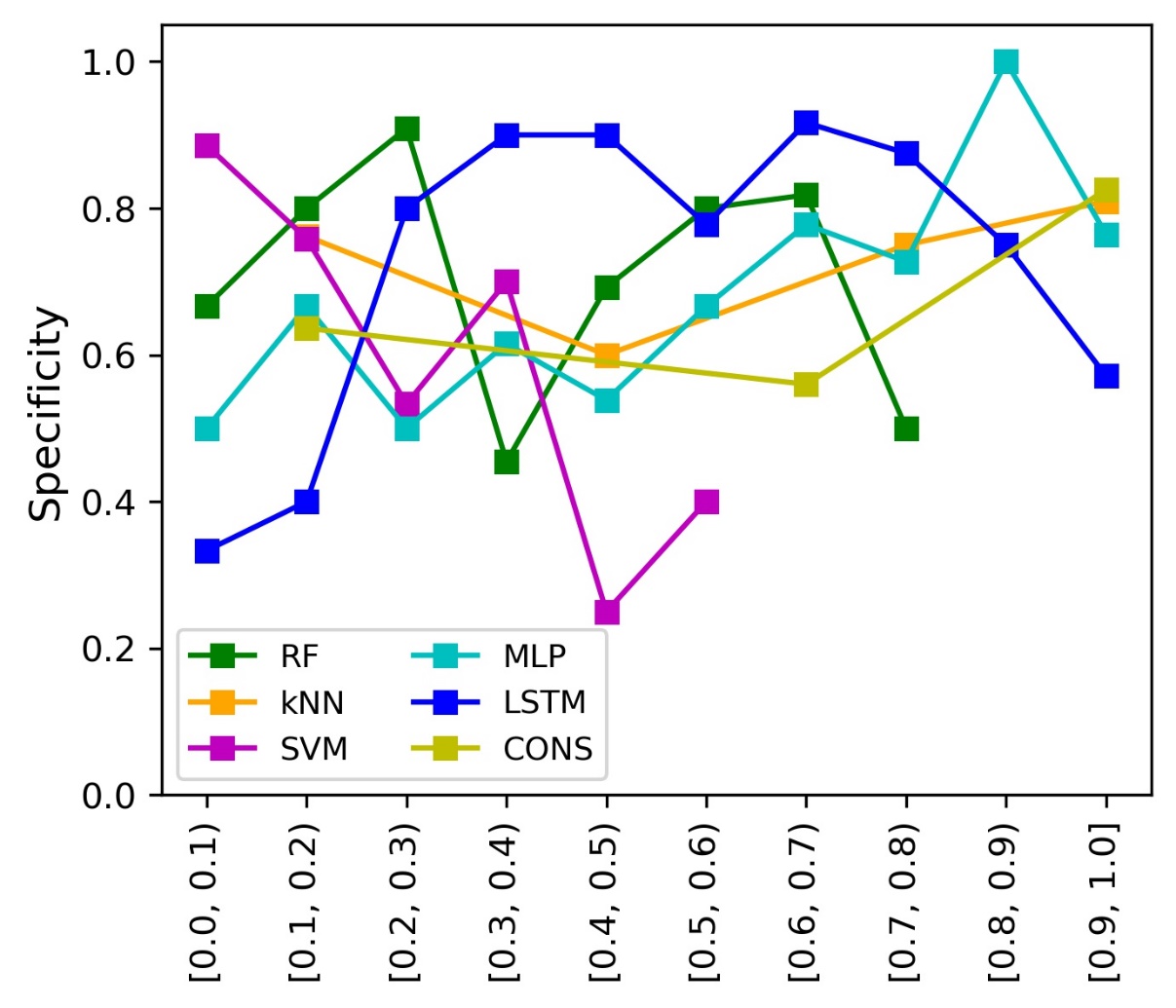
**

**Figure S6.** The specificity of prediction confidence analysis from external validation. The x-axes indicate the ten prediction confidence levels. The specificity values were plotted as the green, orange, magenta, cyan, blue, and yellow lines for Random Forest (RF), k-nearest neighbors (kNN), support vector machine (SVM), Multi-layer Perceptron Classifier (MLP), Long Short-Term Memory (LSTM), and consensus model (CONS), respectively.

**
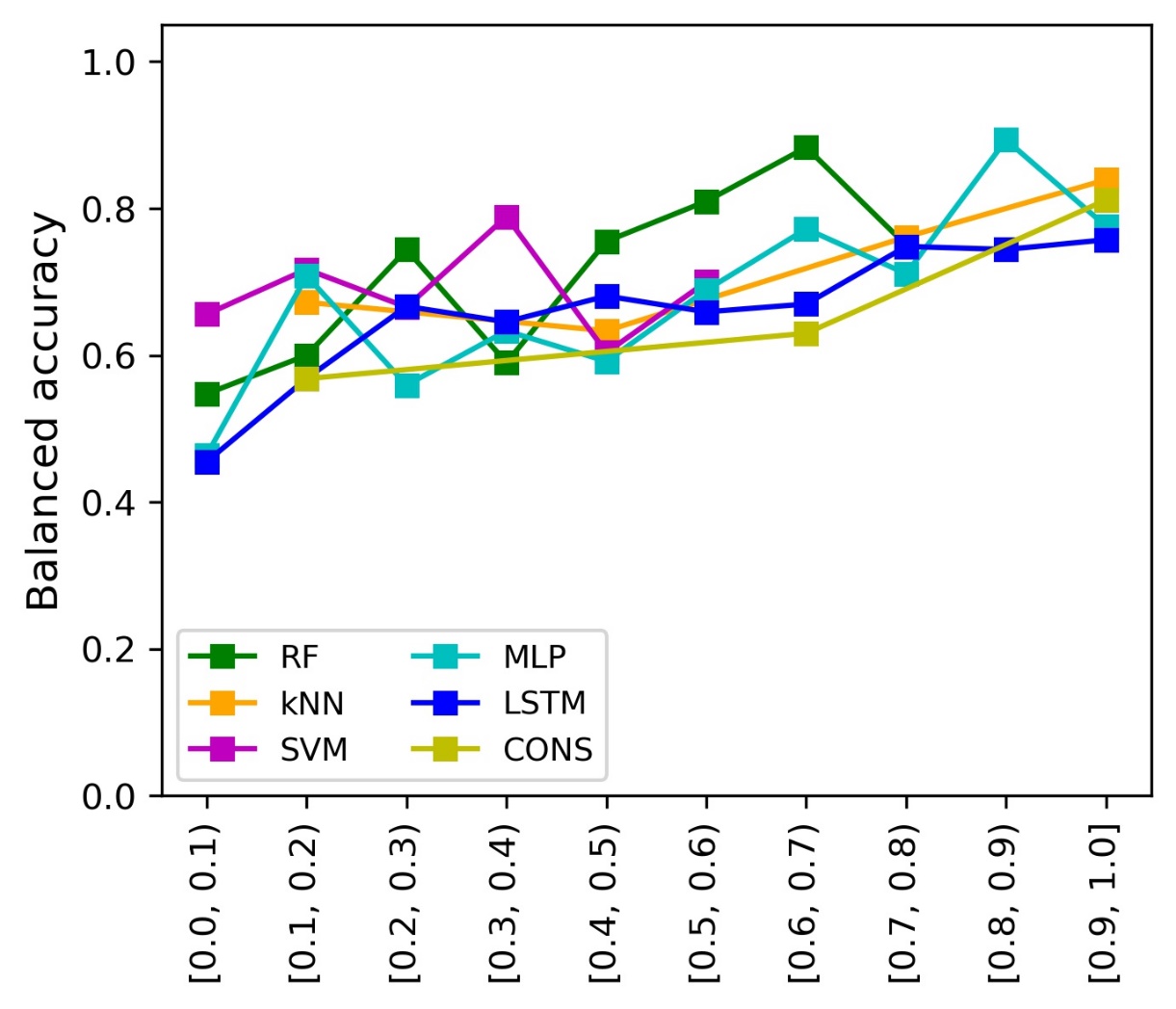
**

**Figure S7.** The balanced accuracy of prediction confidence analysis from external validation. The x-axes indicate the ten prediction confidence levels. The balanced accuracy values were plotted as the green, orange, magenta, cyan, blue, and yellow lines for Random Forest (RF), k-nearest neighbors (kNN), support vector machine (SVM), Multi-layer Perceptron Classifier (MLP), Long Short-Term Memory (LSTM), and consensus model (CONS), respectively.

**
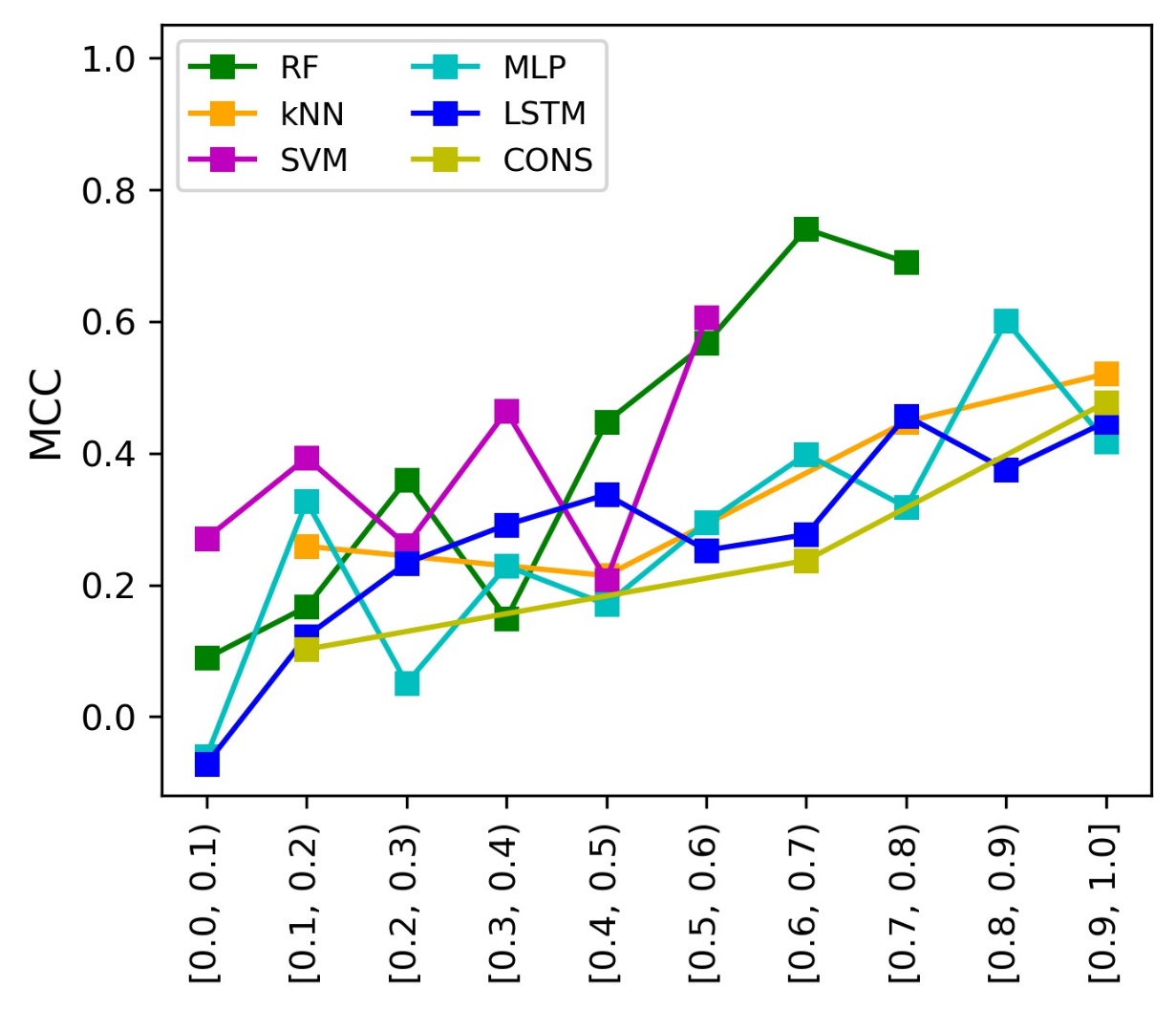
**

**Figure S8.** The MCC of prediction confidence analysis from external validation. The x-axes indicate the ten prediction confidence levels. The MCC values were plotted as the green, orange, magenta, cyan, blue, and yellow lines for Random Forest (RF), k-nearest neighbors (kNN), support vector machine (SVM), Multi-layer Perceptron Classifier (MLP), Long Short-Term Memory (LSTM), and consensus model (CONS), respectively.

**
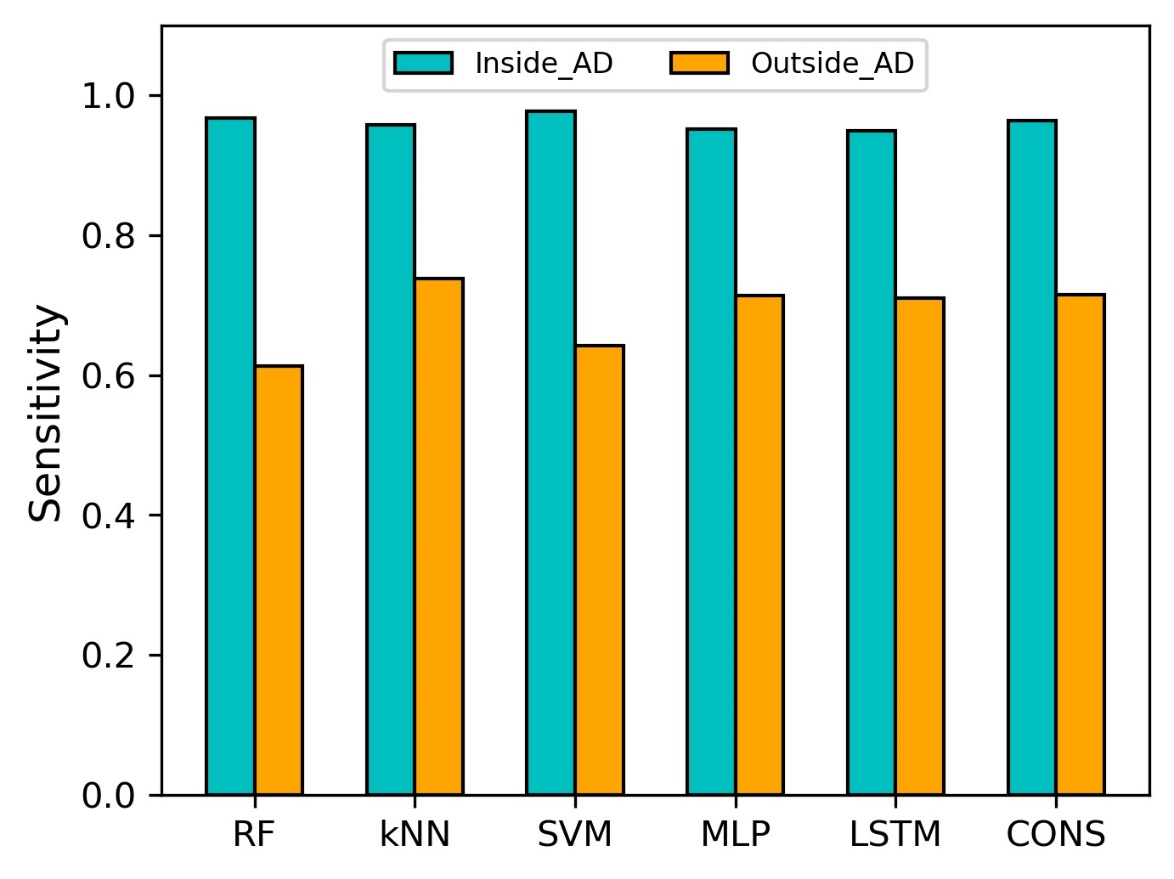
**

**Figure S9.** The sensitivity of applicability domain analysis of cross-validations. The x-axes represent Random Forest (RF), k-nearest neighbors (kNN), support vector machine (SVM), Multi-layer Perceptron Classifier (MLP), Long Short-Term Memory (LSTM), and consensus model (CONS). The bars represent the values of sensitivity from the compounds inside AD in cyan color and outside AD in orange color.

**
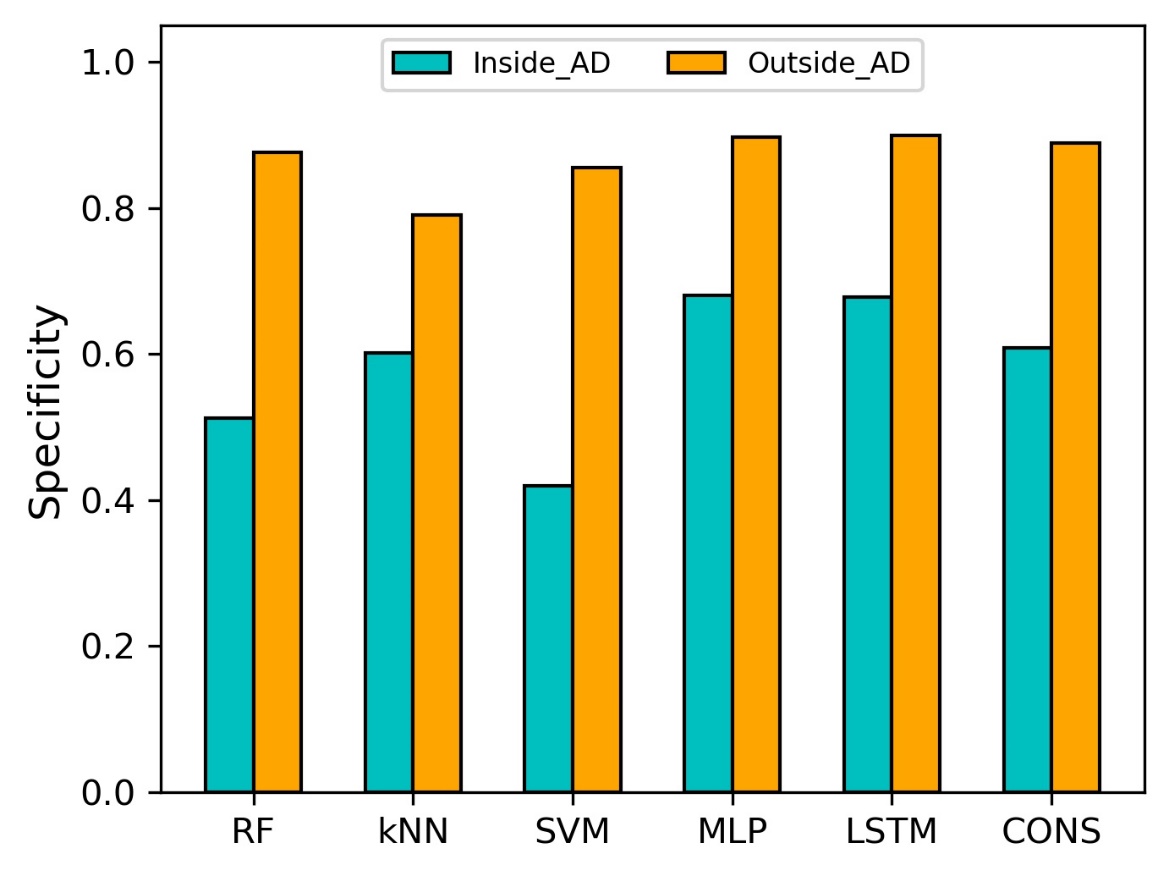
**

**Figure S10.** The specificity of applicability domain analysis of cross-validations. The x-axes represent Random Forest (RF), k-nearest neighbors (kNN), support vector machine (SVM), Multi-layer Perceptron Classifier (MLP), Long Short-Term Memory (LSTM), and consensus model (CONS). The bars represent the values of specificity from the compounds inside AD in cyan color and outside AD in orange color.

**
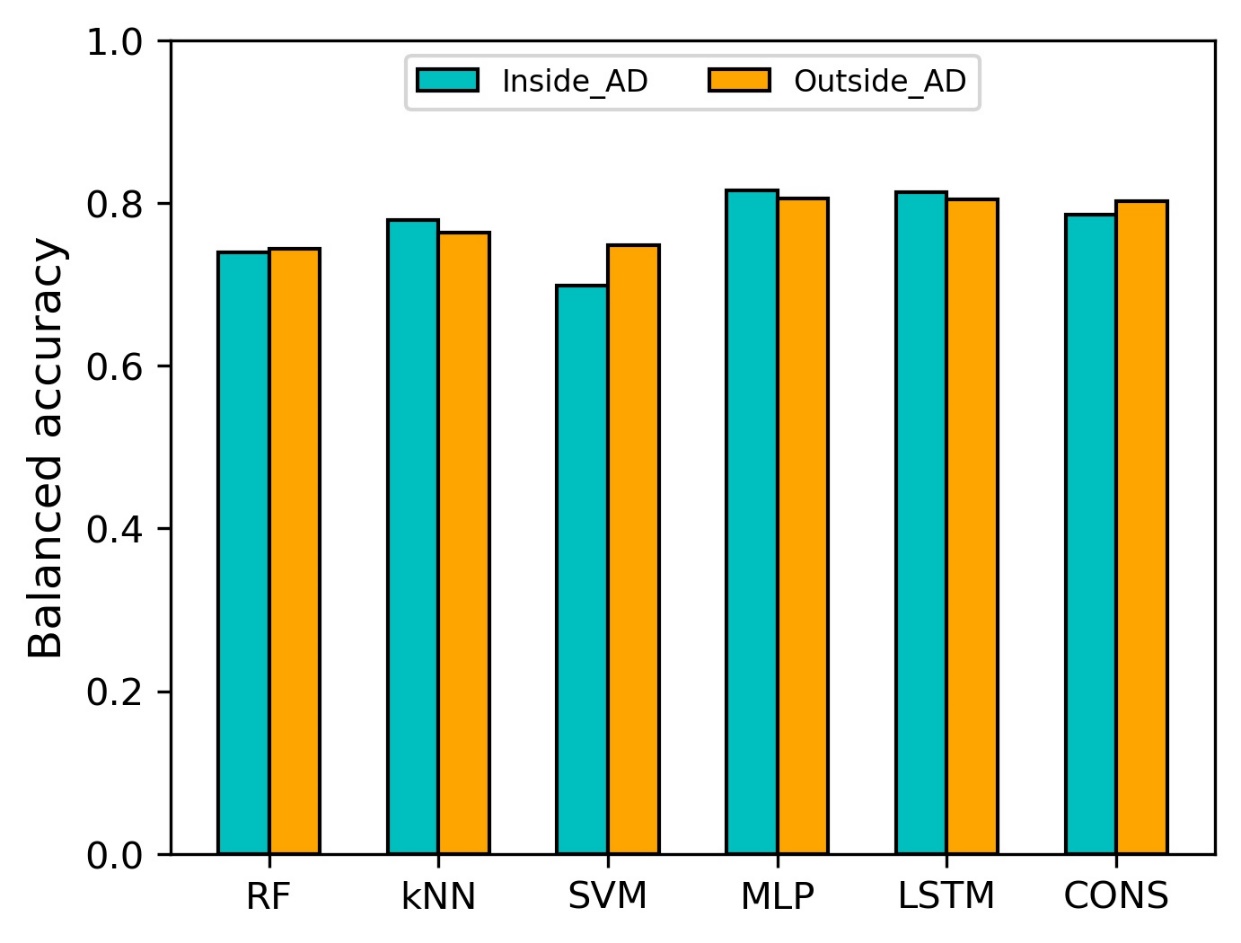
**

**Figure S11.** The balanced accuracy of applicability domain analysis of cross-validations. The x-axes represent Random Forest (RF), k-nearest neighbors (kNN), support vector machine (SVM), Multi-layer Perceptron Classifier (MLP), Long Short-Term Memory (LSTM), and consensus model (CONS). The bars represent the values of balanced accuracy from the compounds inside AD in cyan color and outside AD in orange color.

**
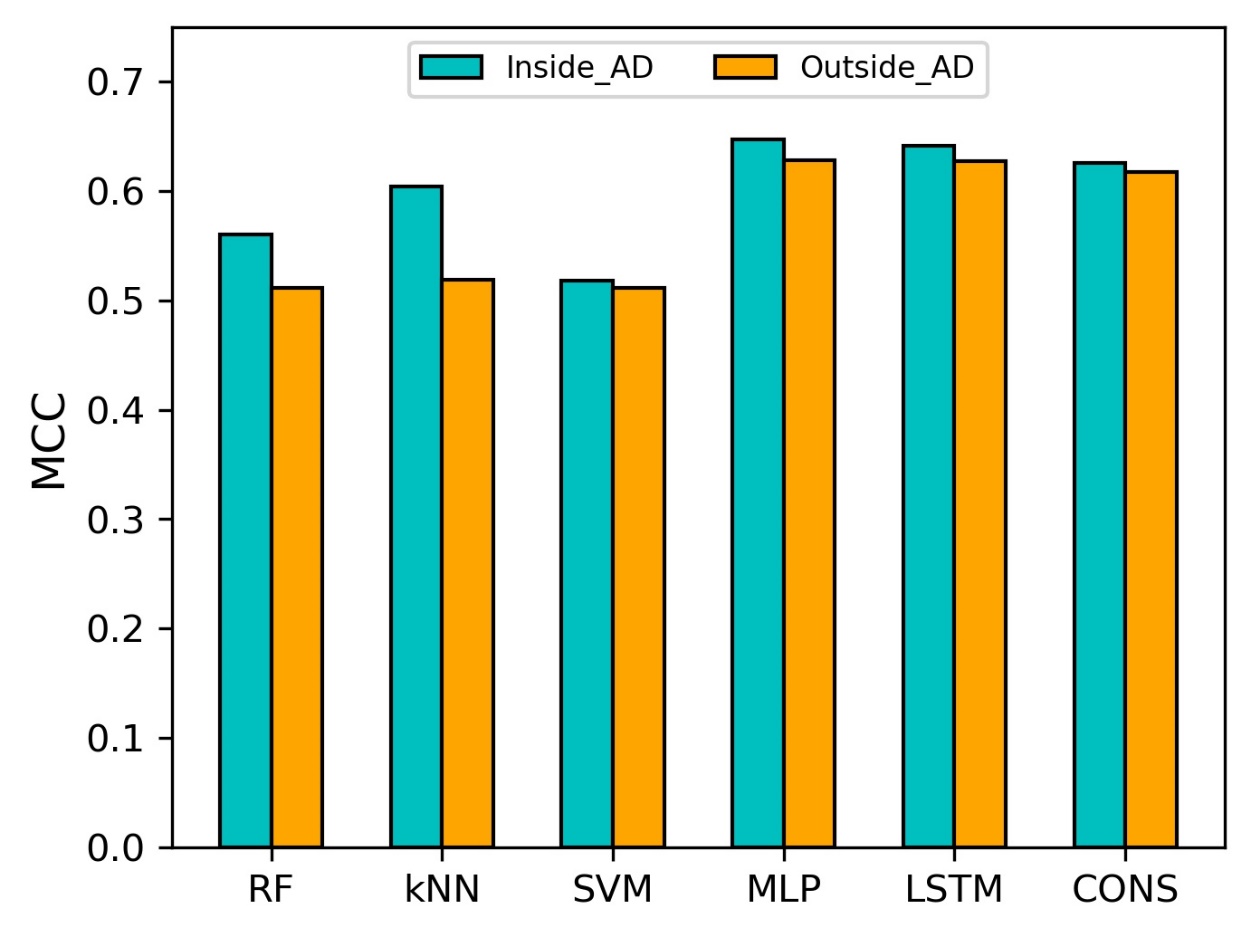
**

**Figure S12.** The MCC of applicability domain analysis of cross-validations. The x-axes represent Random Forest (RF), k-nearest neighbors (kNN), support vector machine (SVM), Multi-layer Perceptron Classifier (MLP), Long Short-Term Memory (LSTM), and consensus model (CONS). The bars represent the values of MCC from the compounds inside AD in cyan color and outside AD in orange color.

**
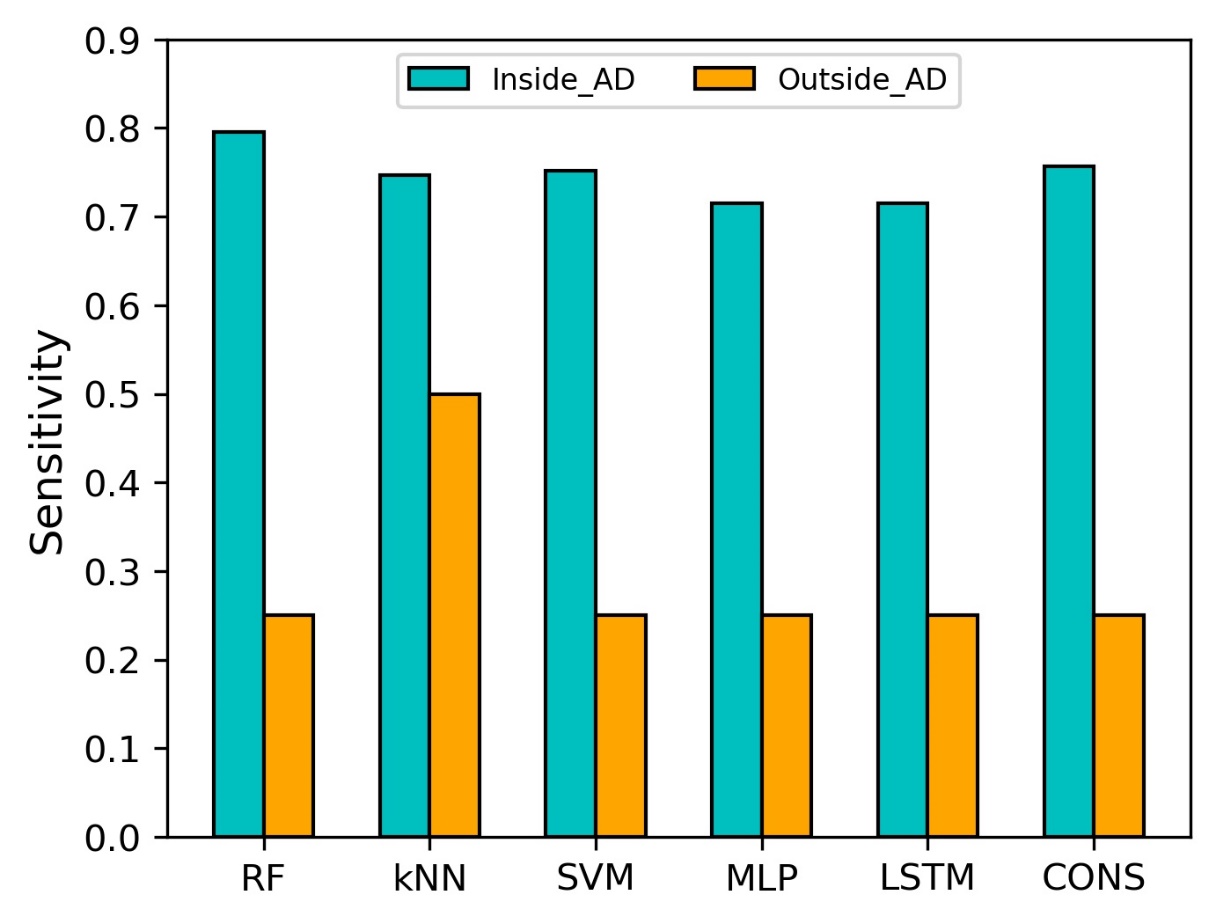
**

**Figure S13.** The sensitivity of applicability domain analysis of external validation. The x-axes represent Random Forest (RF), k-nearest neighbors (kNN), support vector machine (SVM), Multi-layer Perceptron Classifier (MLP), Long Short-Term Memory (LSTM), and consensus model (CONS). The bars represent the values of sensitivity from the compounds inside AD in cyan color and outside AD in orange color.

**
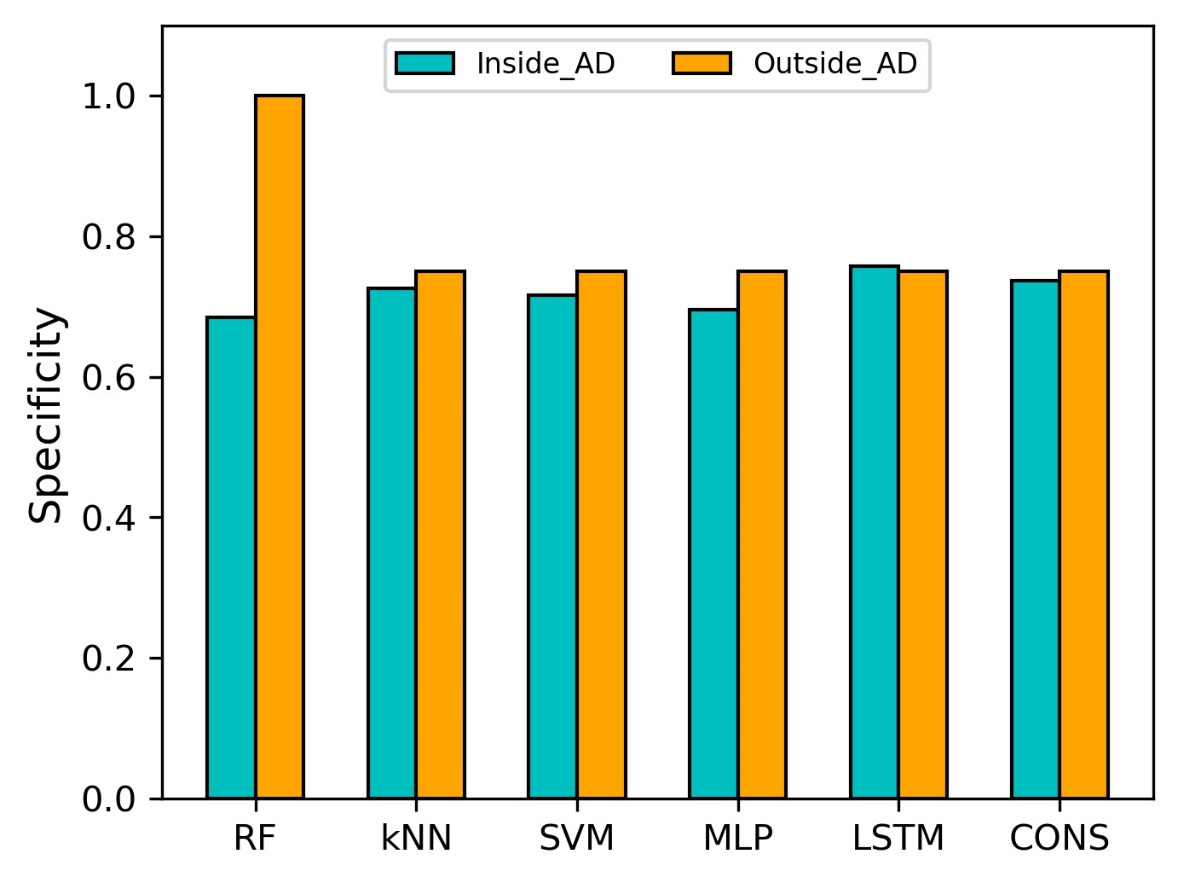
**

**Figure S14.** The specificity of applicability domain analysis of external validation. The x-axes represent Random Forest (RF), k-nearest neighbors (kNN), support vector machine (SVM), Multi-layer Perceptron Classifier (MLP), Long Short-Term Memory (LSTM), and consensus model (CONS). The bars represent the values of specificity from the compounds inside AD in cyan color and outside AD in orange color.

**
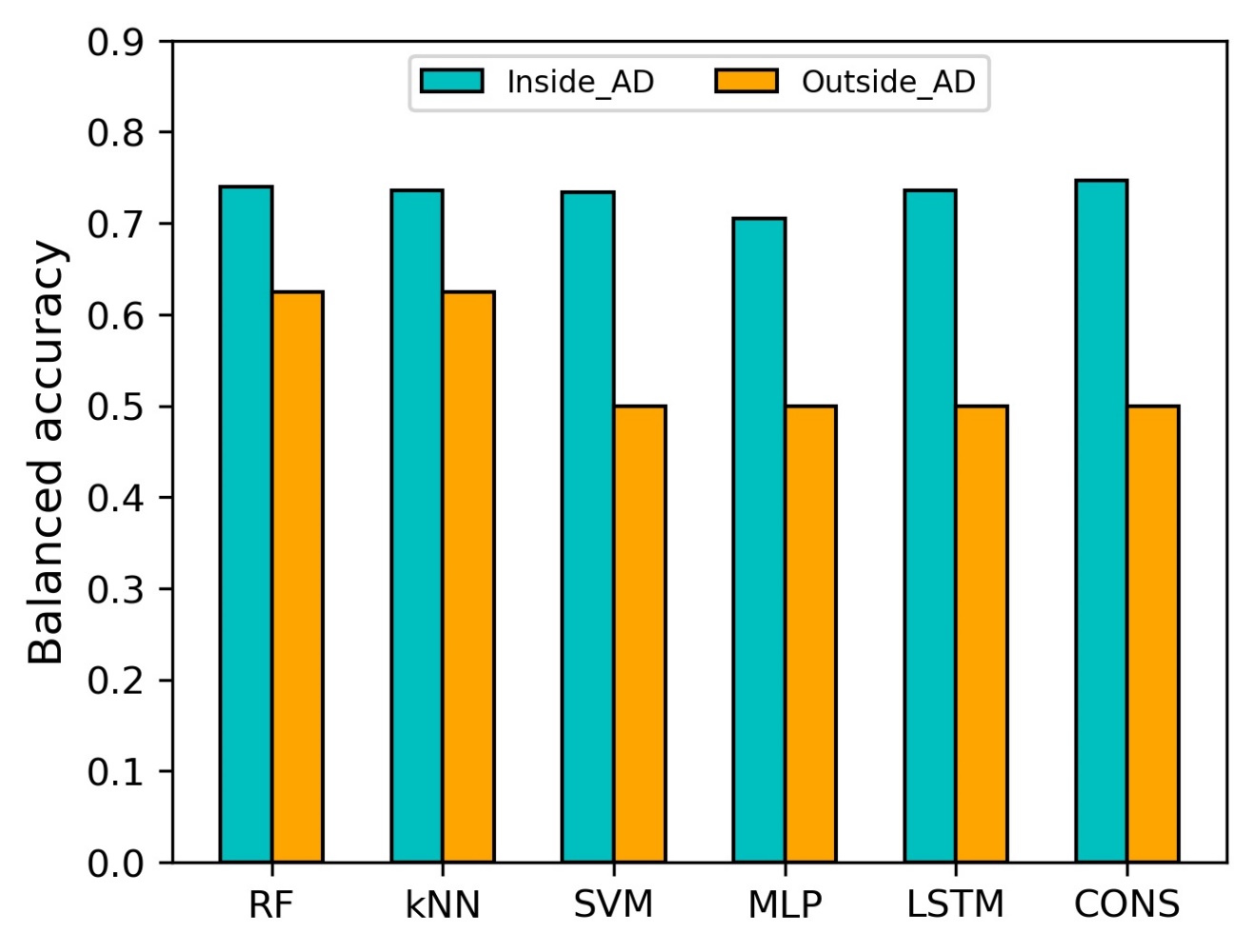
**

**Figure S15.** The balanced accuracy of applicability domain analysis of external validation. The x-axes represent Random Forest (RF), k-nearest neighbors (kNN), support vector machine (SVM), Multi-layer Perceptron Classifier (MLP), Long Short-Term Memory (LSTM), and consensus model (CONS). The bars represent the values of balanced accuracy from the compounds inside AD in cyan color and outside AD in orange color.

**
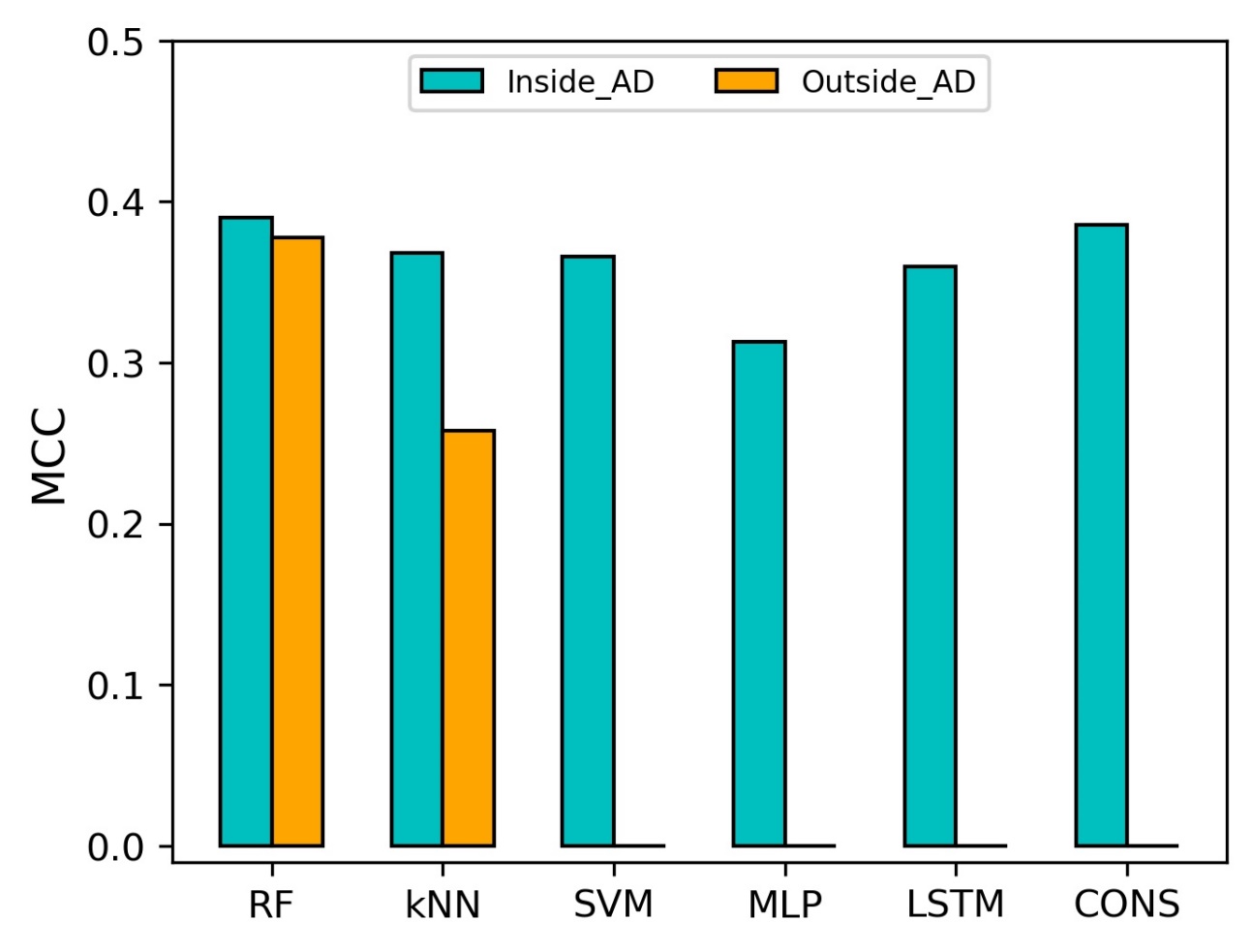
**

**Figure S16.** The MCC of applicability domain analysis of external validation. The x-axes represent Random Forest (RF), k-nearest neighbors (kNN), support vector machine (SVM), Multi-layer Perceptron Classifier (MLP), Long Short-Term Memory (LSTM), and consensus model (CONS). The bars represent the values of MCC from the compounds inside AD in cyan color and outside AD in orange color.


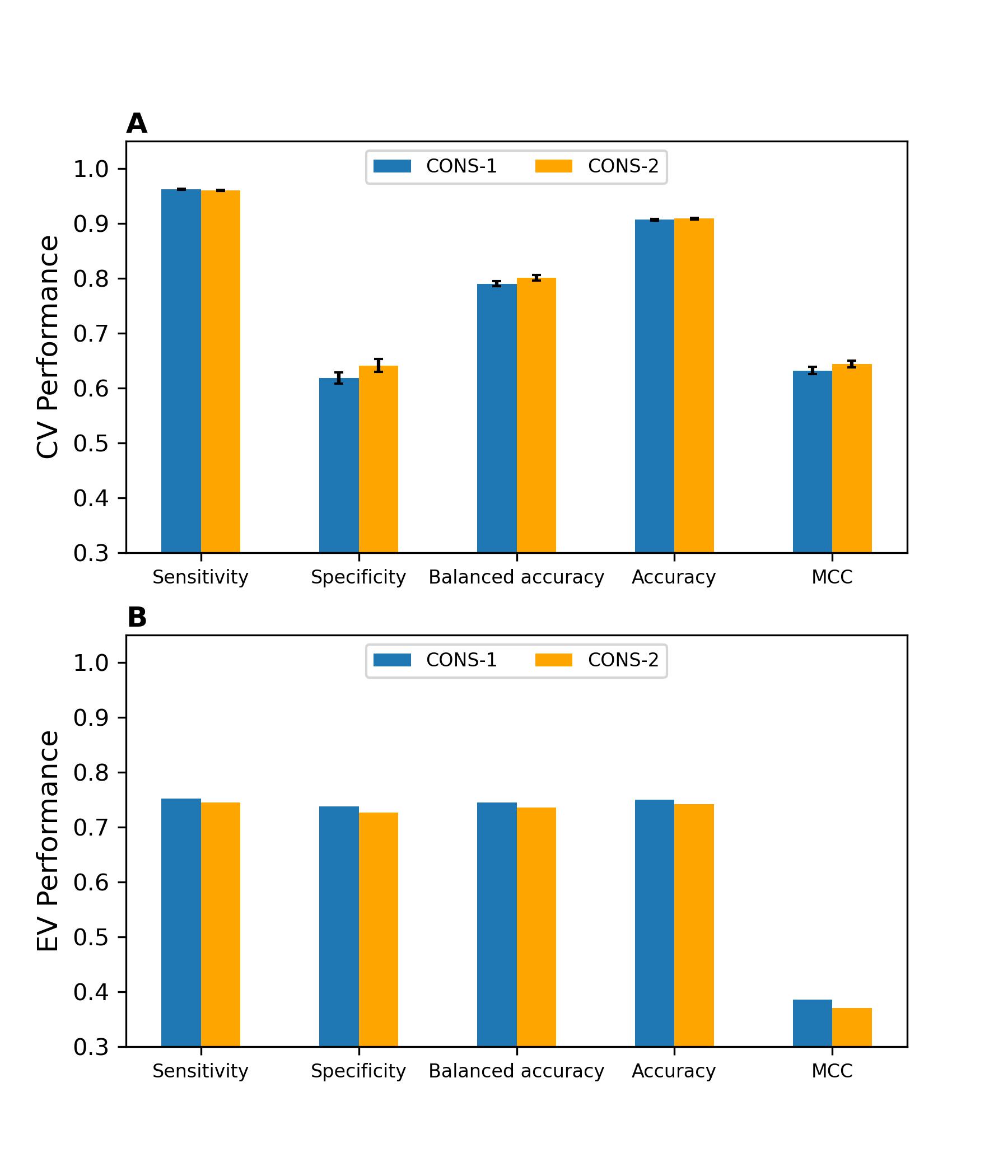


Figure S17. The performance of consensus models from cross-validation (CV) (A) and external validation (EV) (B). The x-axes represent the performance metrics. The bars represent the consensus models from majority voting in blue color (CONS-1) and from average prediction probability in orange color (CONS-2).

**
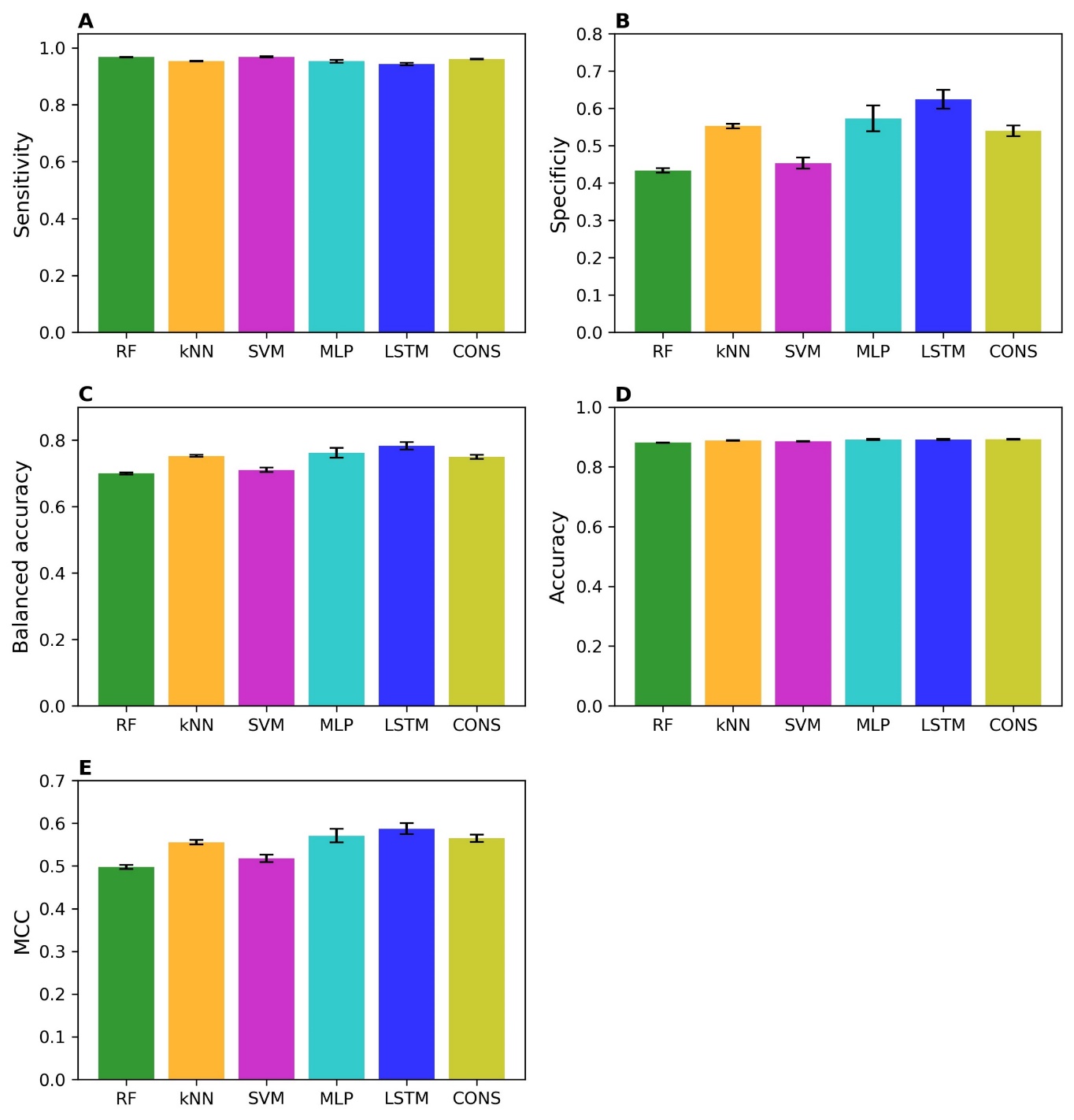
**

**Figure S18**. The performance of cross-validations from stratified selected data. The bars represent the average values and standard deviations of sensitivity (A), specificity (B), balanced accuracy (C), accuracy (D), and MCC (E) from the 50 iterations of cross-validations. The x-axes indicate Random Forest (RF), k-nearest neighbors (kNN), support vector machine (SVM), Multi-layer Perceptron Classifier (MLP), Long Short-Term Memory (LSTM), and consensus model (CONS).

**
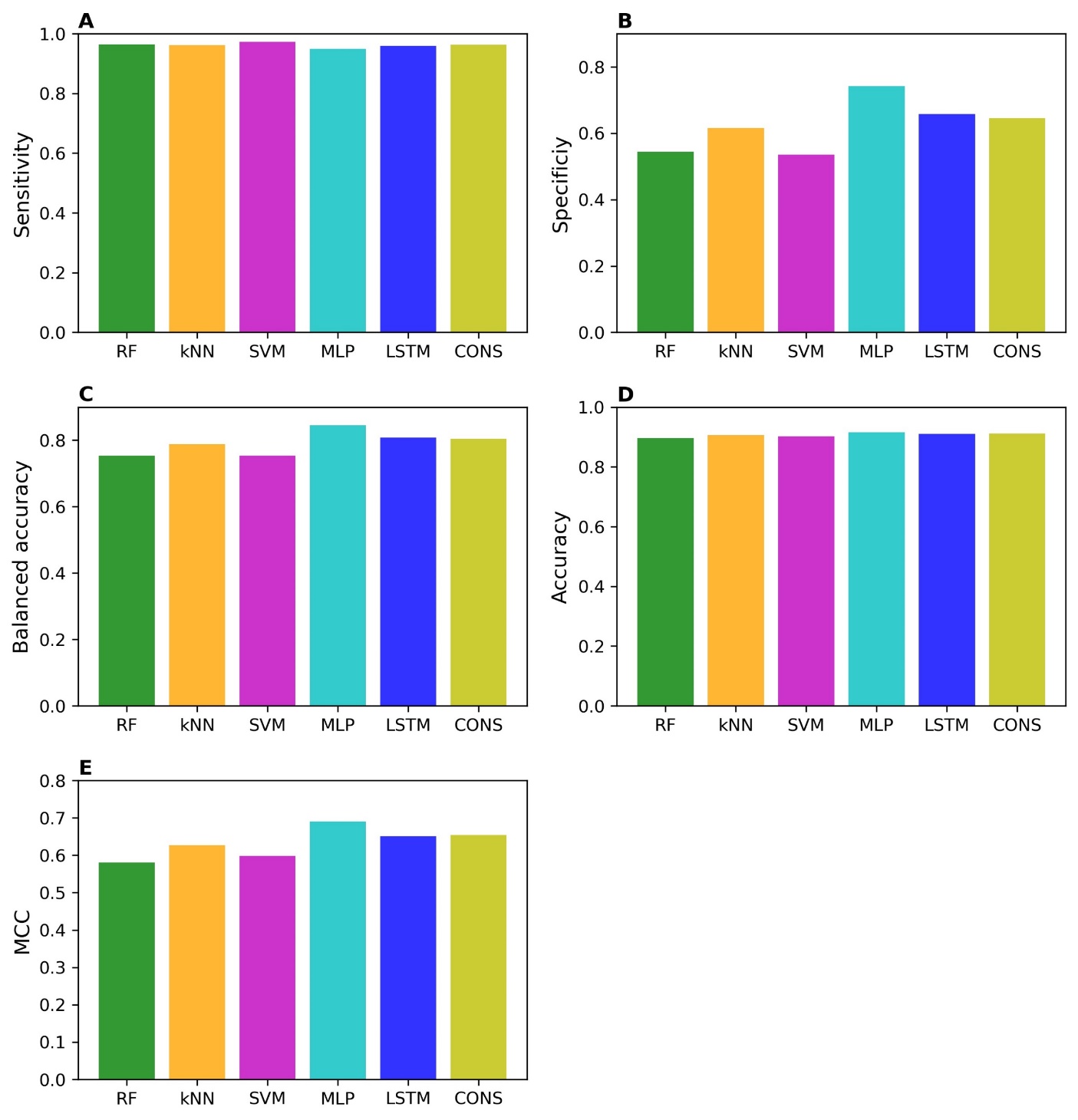
**

**Figure S19**. The performance of external validation from stratified selected data. The bars represent the values of sensitivity (A), specificity (B), balanced accuracy (C), accuracy (D), and MCC (E) from external validation. The x-axes show Random Forest (RF), k-nearest neighbors (kNN), support vector machine (SVM), Multi-layer Perceptron Classifier (MLP), Long Short-Term Memory (LSTM), and consensus model (CONS).
