# Supplementary material for: Developing predictive models for µ opioid receptor binding using machine learning and deep learning techniques
Source: Exp Biol Med (Maywood). 2025 Mar 19;250:10359. doi: 10.3389/ebm.2025.10359 (PMC11961360; doi:10.3389/ebm.2025.10359)
Supplement: Supplementary file 3 [file DataSheet5.docx]

**Supplementary Document**

**Model development and validation using dataset generated from stratified sampling**

We used the stratified sampling approach to generate the training and external validation datasets to keep the same prevalence of MOR binders in both the training and external validation datasets. In details, the new training dataset has 9,987 randomly selected compounds (active/inactive = 8,373/1,614) from 80% of binders from public databases, 80% of non-binders from public databases, 80% of binders from the manuscript, and 80% of non-binders from the manuscript. The rest 2,497 compounds from public databases and the manuscript are used as the new external validation dataset (active/inactive = 2,094/403). The same data preprocessing, model development and evaluation methods were applied to the new training and external validation datasets. The prediction performance results from cross-validations and external validation were included as Supplementary Figures S18 and S19, respectively. The cross-validations performance from the stratified sampling datasets included sensitivity (0.943 – 0.968), specificity (0.433 – 0.624), balanced accuracy (0.700 – 0.784), accuracy (0.881 – 0.892), and MCC (0.497 – 0.587). Compared with the sensitivity (0.949 – 0.976), specificity (0.435 – 0.688), balanced accuracy (0.706 – 0.819), accuracy (0.889 – 0.908), and MCC (0.528 – 0.654), the stratified data split had worse performance of cross-validations. The external validation performance from the stratified sampling datasets included sensitivity (0.949 – 0.972), specificity (0.536 – 0.742), balanced accuracy (0.754 – 0.846), accuracy (0.897 – 0.916), and MCC (0.581 – 0.690). Compared with sensitivity (0.711 – 0.792), specificity (0.697 – 0.758), balanced accuracy (0.704 – 0.745), accuracy (0.709 – 0.776), and MCC (0.315 – 0.399) from external validation, the stratified data sampling improved the performance of external validation, especially the MCC. The stratified sampling generated the training and external validation datasets with the similar part of traditional assays and HTS assays. It is expected that the similar datasets have better performance on external validation. The original training dataset had mainly the traditional assays. Therefore, the model should be suitable to predict traditional assays in the application and may not perform well on the external validation dataset with a different type of assay. It confirmed that every model has limitations based on the data used to train the model.
